# Age, dose, and binding to TfR on blood cells influence brain delivery of a TfR-transported antibody

**DOI:** 10.1186/s12987-023-00435-2

**Published:** 2023-05-11

**Authors:** Rebecca Faresjö, Dag Sehlin, Stina Syvänen

**Affiliations:** Department of Public Health and Caring Sciences, Uppsala University, Uppsala, Sweden

**Keywords:** Brain delivery, Bispecific antibody, Age, Aging, Transferrin receptor

## Abstract

**Background:**

Transferrin receptor 1 (TfR1) mediated brain delivery of antibodies could become important for increasing the efficacy of emerging immunotherapies in Alzheimer's disease (AD). However, age, dose, binding to TfR1 on blood cells, and pathology could influence the TfR1-mediated transcytosis of TfR1-binders across the blood–brain barrier (BBB). The aim of the study was, therefore, to investigate the impact of these factors on the brain delivery of a bispecific TfR1-transported Aβ-antibody, mAb3D6-scFv8D3, in comparison with the conventional antibody mAb3D6.

**Methods:**

Young (3–5 months) and aged (17–20 months) WT and tg-ArcSwe mice (AD model) were injected with ^125^I-labeled mAb3D6-scFv8D3 or mAb3D6. Three different doses were used in the study, 0.05 mg/kg (low dose), 1 mg/kg (high dose), and 10 mg/kg (therapeutic dose), with equimolar doses for mAb3D6. The dose-corrected antibody concentrations in whole blood, blood cells, plasma, spleen, and brain were evaluated at 2 h post-administration. Furthermore, isolated brains were studied by autoradiography, nuclear track emulsion, and capillary depletion to investigate the intrabrain distribution of the antibodies, while binding to blood cells was studied in vitro using blood isolated from young and aged mice.

**Results:**

The aged WT and tg-ArcSwe mice showed significantly lower brain concentrations of TfR-binding [^125^I]mAb3D6-scFv8D3 and higher concentrations in the blood cell fraction compared to young mice. For [^125^I]mAb3D6, no significant differences in blood or brain delivery were observed between young and aged mice or between genotypes. A low dose of [^125^I]mAb3D6-scFv8D3 was associated with increased relative parenchymal delivery, as well as increased blood cell distribution. Brain concentrations and relative parenchymal distribution of [^125^I]mAb3D6-scFv8D6 did not differ between tg-ArcSwe and WT mice at this early time point but were considerably increased compared to those observed for [^125^I]mAb3D6.

**Conclusion:**

Age-dependent differences in blood and brain concentrations were observed for the bispecific antibody mAb3D6-scFv8D3 but not for the conventional Aβ antibody mAb3D6, indicating an age-related effect on TfR1-mediated brain delivery. The lowest dose of [^125^I]mAb3D6-scFv8D3 was associated with higher relative BBB penetration but, at the same time, a higher distribution to blood cells. Overall, Aβ-pathology did not influence the early brain distribution of the bispecific antibody. In summary, age and bispecific antibody dose were important factors determining brain delivery, while genotype was not.

**Supplementary Information:**

The online version contains supplementary material available at 10.1186/s12987-023-00435-2.

## Introduction

An increasing number of antibodies for central nervous system (CNS) diseases are studied in clinical trials [1, 2], and two amyloid-beta (Aβ) targeting antibodies have recently gained conditional approval from the FDA, aducanumab (Aduhelm^®^) and lecanemab (Leqembi^®^). They are the first disease-modifying treatments for Alzheimer's Disease (AD) [3].

However, antibodies are, to a large degree, restricted from entering the brain due to the tightly controlled passage of large molecules across the blood–brain barrier (BBB). Generally, less than 0.1% of the injected dose of an IgG antibody reaches the brain [4–11]. The highest dose (10 mg/kg) of lecanemab, administered 2 times per month, was most efficient in reducing brain Aβ burden and slow cognitive decline, indicating that high brain concentration of antibody is important for successful treatment [12, 13]. Different receptors at the BBB have been utilized to increase brain concentrations further for active transcytosis of antibodies and large protein-based drugs across the brain capillary endothelial cells (BCECs) [14]. The transferrin receptor (TfR) was the earliest described and still a widely used target for BBB-shuttles [15]. In numerous preclinical studies, TfR has consistently shown high efficiency in delivering proteins across the BBB [11, 16–20]. Presently, bispecific proteins are also entering clinical use; for example, an enzyme replacement therapy for Hunters Syndrome utilizing the TfR for receptor-mediated brain delivery was approved in Japan in 2021, providing clinical proof-of-concept [21–23]. Thus, there is a clear rationale for using TfR-mediated delivery of antibody and large protein drugs to increase intrabrain concentrations and thereby improve treatment efficacy and, at the same time, avoid administration of high doses associated with side effects and high costs.

At the BBB, TfR allows transferrin-bound iron to transcytose across the endothelial cells to the brain parenchyma. The TfR expression is especially high in the capillaries of the brain [24]. TfR is a 190 kDa transmembrane glycoprotein, with two subtypes, TfR1 and TfR2, as well as a soluble form of the cleaved TfR1 present in blood serum, sTfR [25]. TfR2 has a lower affinity for transferrin, and the expression is mainly limited to the liver and early erythroid cells [26]. TfR1 is not only expressed in the brain endothelial cells but by most cells in the body. It is especially high in cells that require a large amount of iron, for example, tumor cells, proliferating cells of the bone marrow, and immature red blood cells (reticulocytes) [27]. Reticulocytes are released from the bone marrow, and in rodents, also from the spleen, to the circulation to mature into red blood cells [28]. The reticulocyte TfR1 levels decrease during maturation and reach very low levels on the cell surface in mature red blood cells [29].

Only a few studies have investigated how brain TfR1 levels or TfR1-transported antibodies are affected in AD pathology or by aging. In the human brain, two different studies have shown similar TfR1-levels in AD, in early AD, and in healthy age-matched controls [30, 31]. Similarly, in mice, studies show that amyloid-precursor protein (APP)-transgenic mice have similar TfR1 levels as WT controls [30, 31]. Also in agreement with this, two TfR1-transported antibodies, the TfR1-antibody Ri7 and the low TfR1-affinity bispecific antibody anti-TfR^D^/BACE1, both showed similar brain concentrations in WT and AD-model mice [30, 31]. Regarding age differences, a study in APP-knockout mice showed decreased TfR1 protein levels at 22 months, compared to 8-month-old APP-KO mice, and also suggested a trend of decreased TfR1 levels in WT mice at old age [32]. In contrast, Bourassa et al. did not observe significant differences between 12 months old and 18–22 months old WT mice, in TfR1 levels or Ri7 brain uptake, while ages were not compared directly in the study by Bien-Ly et al. [30, 31]. Moreover, physiological functions at the BBB could be affected by old age. For example, protein uptake by transcytosis has been described as impaired in an extensive study by Yang et al., which also indicated that TfR expression is affected in aged mice [33]. Therefore, there is a need for more studies systematically investigating TfR1 and TfR1-antibody brain delivery in aging, in parallel to AD pathology.

The impact of binding to blood cell-expressed TfR1 on brain delivery of bispecific antibodies has not been studied widely, even though this peripheral binding is generally addressed as a potential source for side effects. For example, TfR1 antibodies, at least when administered at high doses, have been shown to deplete the amount of circulating reticulocytes in mice [34]. The percentage of circulating reticulocytes in peripheral blood is approximately 1.1% in mice, while in humans it is significantly lower, around 0.0–0.2% [34]. Thus, this effect may be considered less likely to occur in humans [35]. Aside from contributing to blood cell toxicity, binding to TfR1 in blood could influence brain delivery due to a reduced fraction of unbound TfR1-binder concentration available for brain delivery. This aspect has perhaps been disregarded as most studies use high doses (≥ 10 mg/kg) that are likely to saturate TfR1 in blood, and thus, ensure a large proportion of unbound antibody in the plasma. However, the "free drug hypothesis" is a well-established concept for small molecule drugs and states that only the fraction of unbound drug in plasma is available to exhibit pharmacological effects by interaction with the drug target [36]. Likewise, for TfR1 antibodies, binding to blood cells, or even to sTfR1 in plasma, could impede the availability of antibody in plasma for transcytosis at the BBB. Especially at lower doses, the sequestering of TfR1-binding antibodies in blood could impede delivery across the BBB. Moreover, TfR1-antibodies are subjected to target-mediated clearance, and could alter the expression levels of TfR1, potentially leading to changed pharmacokinetics of the antibody. A bispecific antibody that in addition to its primary targets also binds to TfR1 often displays a shorter half-life in blood compared to its conventional monospecific IgG version [5, 20]. We have previously observed that tracer doses (< 0.1 mg/kg) of i.v. administered bispecific antibodies that bind both murine (m)TfR1 and Aβ, are present to a high degree in the blood cell pellet after centrifugation of whole blood samples [37, 38]. This indicated that the majority of the bispecific antibodies were associated with blood cells rather than with plasma at this dose.

The aim of the present study was to investigate the impact of age, dose, pathology (presence of Aβ), and binding to mTfR1 expressed by blood cells on brain delivery of a bispecific antibody engineered to enter the brain via mTfR1-mediated transcytosis.

## Methods

### Animals

Wild-type (WT) C57/Bl6 mice and transgenic (tg)-ArcSwe mice maintained on a C57/Bl6 background were used for the study. Mice were either 3–5 months old, i.e., the 'young' group, or 17–20 months old, i.e., the 'aged' group. Tg-ArcSwe mice harbor the Arctic APP mutation (APP E693G), which leads to the production of Aβ that readily aggregates, and the Swedish APP mutation (APP KM670/671NL), which results in over-production of Aβ [39, 40]. The tg-ArcSwe model is characterized by high levels of aggregated Aβ at an early age, forming dense-core plaques when animals are 6–7 months old, and with pronounced pathology after 12 months [39]. Thus, the young tg-ArcSwe group do not yet display plaque pathology, nor vascular plaques—however it is likely that they do produce aggregated species of Aβ in the brain. The mice had free access to food and water and were housed in rooms with controlled temperature and humidity in an animal facility at Uppsala University. Only female mice were included to reduce the number of factors studied, and thereby the number of animals used. The Uppsala Country Animal Ethics board (Application number 5.8.18–20401/2020) approved all experimental procedures following the legislation and regulations of the Swedish Animal Welfare Agency and European Communities Council Directive of 22 September 2010 (2010/63/EU).

### Antibodies, doses and study design

**Fig. 1:**
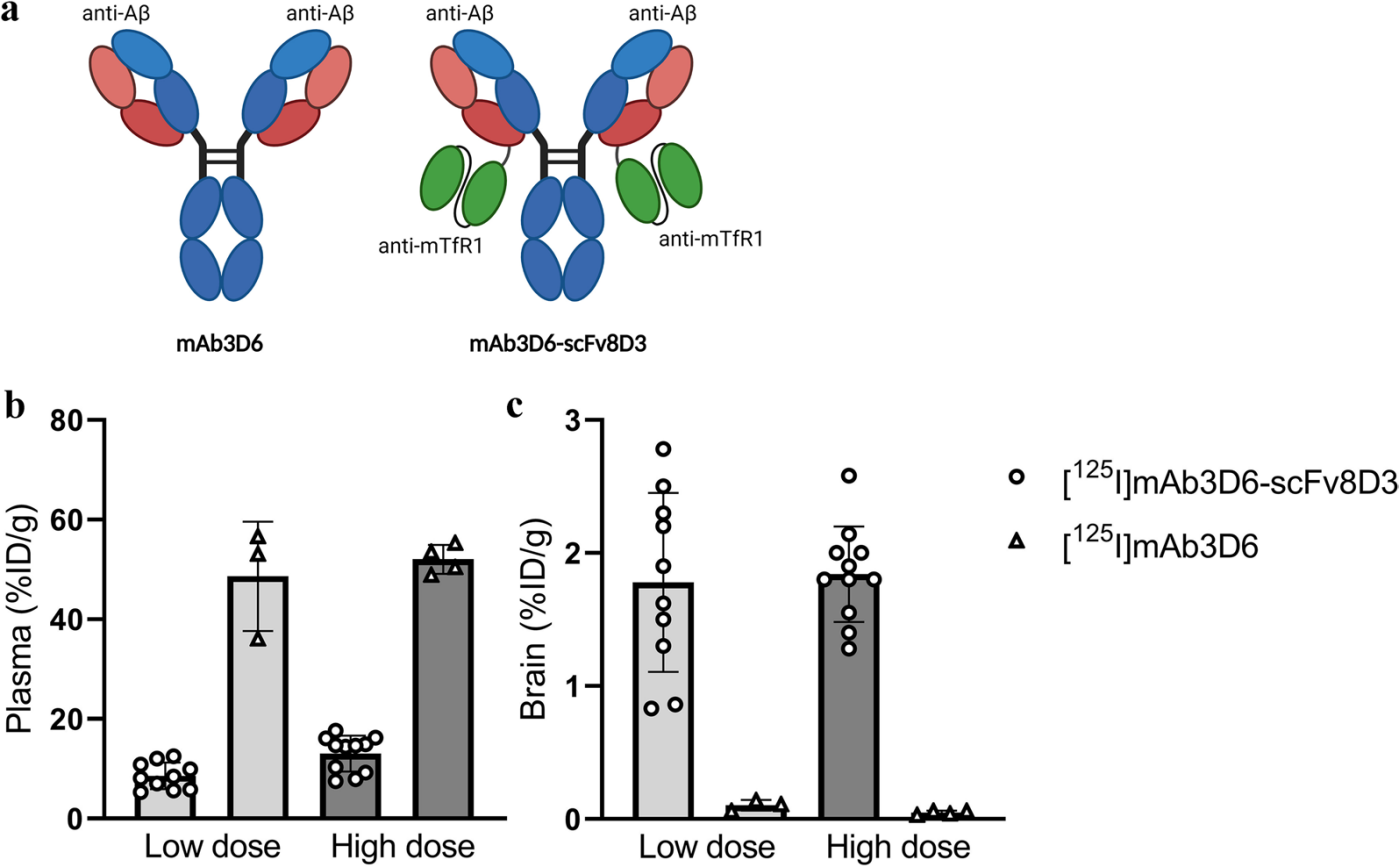
**a** The antibodies mAb3D6 and mAb3D6-scFv8D3 **b** %ID/g plasma and **c** %ID/g brain at 2 h after injection of [^125^I]mAb3D6-scFv8D3 and [^125^I]mAb3D6 at low and high dose

The bispecific antibody mAb3D6-scFv8D3, containing two mTfR1-binding moieties (scFv8D3) based on the mTfR1 antibody 8D3, was compared with the conventional IgG antibody mAb3D6, which lacks TfR binding (Fig. 1a) [7, 41, 42]. Thus, both antibodies contained 3D6, the murine version of the antibody Bapineuzumab that targets the N-terminal end of the Aβ-protein [37, 43–45]. Both the conventional and the bispecific antibody were recombinantly produced in-house as previously described, and a single batch of respective antibody was used for all animal experiments [46]. Two equimolar doses were studied for bispecific mAb3D6-scFv8D3 and mAb3D6: a tracer dose of 0.2 nmol/kg, referred to as low dose (0.05 mg/kg or 0.036 mg/kg, respectively) and a low therapeutic dose, here referred to as the high dose; 5 nmol/kg (1 mg/kg or 0.71 mg/kg respectively). The two antibodies were administered to young and aged WT and tg-ArcSwe mice in a low and a high dose (Table 1). An additional WT group injected with a high therapeutic dose (10 mg/kg) of the bispecific antibody was also included (Table 1).

**Table 1:** Number of mice (n) for each subgroup

[^125^I]mAb3D6-scFv8D3				[^125^I]mAb3D6			Naïve
*WT*	*Low dose*	*High dose*	*Therapeutic dose*	*WT*	*Low dose*	*High dose*	*In vitro blood study/Western blot*
Young	10	11	5	Young	3	4	10
Aged	12	13		Aged	4	8	10
*Tg-ArcSwe*	*Low dose*	*High dose*		*Tg-ArcSwe*	*Low dose*	*High dose*	
Young	7	7		Young	3	4	
Aged	5	7		Aged	4	5	

Total n = 132

### Radiochemistry

The antibodies mAb3D6-scFv8D3 and mAb3D6 were radiolabeled with iodine-125 by the Chloramine-T method [47]. Briefly, 30 μg of mAb3D6-scfv8D3 or mAb3D6 was mixed with 15 ± 3 MBq and 12 ± 2 MBq, respectively, of ^125^I stock solution (Perkin-Elmer Inc Waltham, MA, USA), and 5 μg Chloramine-T (Sigma Aldrich, Stockholm, Sweden) to a total volume of 110 μl. The reaction was incubated for 90 s at room temperature and quenched by the addition of 10 μg of sodium metabisulfite (Sigma Aldrich). The radiolabeled proteins were purified from free iodine using a PBS-equilibrated Zeba spin desalting column (7 K MWCO, 0.5 mL, 89,882, ThermoFischer). The molar activity after radiolabelling was 74.7 ± 14.2 MBq/nmol and 49.3 ± 6.25 MBq/nmol for [^125^I]mAb3D6-scFv8D3 and [^125^I]mAb3D6, respectively.

For the therapeutic dose, 100 μg bispecific antibody was radiolabeled with 12.3 MBq of ^125^I stock solution, then supplemented with 250 μg cold antibody, resulting in a molar activity of 0.97 MBq/nmol.

For the in vitro blood study, 8–10 μg of mAb3D6-scFv8D3 was used for radiolabelling, according to the above description, but using half of the total volume. The molar activity was in this case 55.7 ± 11.2 MBq/nmol after radiolabeling.

### Ex vivo study of ^125^I-labeled antibodies

Mice were injected by the tail vein with [^125^I]mAb3D6-scFv8D3 (0.48 ± 0.19 MBq) or with [^125^I]mAb3D6 (0.50 ± 0.25 MBq). After 2 h, mice were anesthetized and terminal blood samples were taken from the heart followed by transcardial perfusion with 40 mL NaCl for 2.5 min. The brain was dissected into three parts; the right hemisphere, the left cerebellum, and the left hemisphere cerebrum. These samples were immediately snap-frozen on dry ice. The blood samples were centrifuged in heparinized tubes and plasma was carefully separated from the blood pellet. The spleen was also isolated from all animals. The radioactivity in all samples was measured in a γ-counter (2480 WizardTM, Wallac Oy PerkinElmer, Turku, Finland).

Radioactivity in the samples is reported as the percent of injected radioactivity dose per gram tissue without (%ID/g, Eq. 1) or with correction for body weight (bw) of the animal (%ID/g(bw), Eq. 2). Radioactivity in the spleen was quantified as injected dose per organ (%ID) (Eq. 3).

1 $$\mathrm{\%}\frac{\mathrm{ID}}{\mathrm{g}}= \frac{\mathrm{measured \, radioactivity \, per \, gram \, tissue}}{\mathrm{total \, injected \, radioactivity}} \times 100$$

2 $$\mathrm{\%}\frac{\mathrm{ID}}{\mathrm{g}}\left(\mathrm{bw}\right)= \frac{\mathrm{measured \, radioactivity \, per \, gram \, tissue}}{\mathrm{total \, injected \, radioactivity \, per \, gram \, animal}} \times 100$$

3 $$\mathrm{\%ID }= \frac{{\mathrm{Radioactivity}}_{sample}}{\mathrm{Injected \, radioactivity}} \times 100$$

Important to keep in mind, is that all of the above equations represent *dose corrected* concentrations of antibody, if not otherwise stated. This means the dose corrected concentration can be the same after administration of a low and a high dose. In fact, at linear pharmacokinetics, i.e. when there are no processes involving saturation or blocking, the dose corrected concentration should be the same regardless of dose. However, the absolute concentration is in general higher for a high dose compared to a low dose. The use of dose corrected concentrations enables direct comparison of distribution between low and high doses.

### Ex vivo autoradiography

The right brain hemispheres isolated from mice injected with ^125^I-labeled antibody were cryosectioned sagitally (20 μm) (Cryostar NX70, ThermoFischer) and mounted on Superfrost Plus glass slides (Menzel Gmboltion, Braunschweigh, Germany). Duplicate sections from each animal together with a standard of ^125^I with known radioactivity were exposed to a phosphor imaging plate for 7 days. The plates were scanned in an Amersham Typhoon Biomolecular Imager (Cytiva) with the emission filter IP BP390 at 25 μm/pixel. The generated digital image brightness and contrast were adjusted in ImageJ. The radioactivity standards were used to normalize intensities for images. The whole brain was used as a region of interest for the quantifications in ImageJ.

### Capillary depletion

Capillary depletion was performed on selected animals, as previously described, with slight modifications [15, 37, 48]. In short, the left cortex was isolated immediately after transcardial perfusion. The cortex was weighed and measured in a γ-counter for 1 min on ice before homogenization in 0.4 ml cold physiological buffer (10 mM HEPES, 141 mM NaCl, 4 mM KCl, 2.8 mM CaCl2, 1 mM MgSO4, 1 mM NaH2PO4, 10 mM D-glucose adjusted to pH 7.4) with 6 strokes in an ice cold Dounce homogenizer. Thereafter, 0.8 ml of 30% Ficoll 400 (Sigma Aldrich) was added followed by an additional stroke. The homogenate was transferred to a 15 ml Falcon tube, followed by centrifugation at 5200 × g for 20 min at 4 °C. The centrifugation resulted in a parenchymal supernatant with a layer of fat, and a capillary-enriched pellet that was carefully separated from the supernatant. The activity in each fraction was measured in a γ-counter (PerkinElmer). The relative distribution (%) of the radioactive signal was determined for each fraction:

4 $$\mathrm{\% }= \frac{{\mathrm{Radioactivity}}_{fraction}}{{\rm{Radioactivity}}_{Pellet}+ {\rm{Radioactivity}}_{Supernatant}} \times 100$$

Or normalized to the injected dose (%ID) in MBq (Eq. 3).

### Immunofluorescence with vascular marker CD31 and Aβ-staining

Sagittal cryosections were fixed in ice-cold MeOH for 10 min. Thereafter, the sections were washed for 2 × 5 min in PBS. CD31 staining on WT sections and double CD31/Aβ40 staining was done on selected tg-ArcSwe brain sections, by the following procedure:

The sections were blocked for 1 h with 5% Normal Goat Serum, followed by a wash in PBS. The primary antibodies rat-α-mouse CD31 (BD, #553,370) and rabbit-α-Aβ40 (Agrisera, Umeå, Sweden) were applied to the sections which were incubated overnight at 4 °C. After incubation, the sections were washed in PBS and secondary antibody goat-anti-rat (Alexa 647) or goat-anti-rabbit (Alexa 488), was added for 1 h with slow shaking, followed by a PBS wash. The sections were stored in PBS until the nuclear track emulsion procedure (described below) was performed on the same day.

### Nuclear track emulsion autoradiography

Nuclear track emulsion autoradiography (NTE) experiments were done in darkness as previously described [5]. In brief, ILFORD K5 emulsion was prepared in a 40 °C water bath according to the manufacturer's instructions. The immunofluorescently stained sections were dipped in the emulsion for 5 s and left to air dry for 2 h, followed by 4 weeks of incubation in darkness at 4 °C. The sections were developed according to the manufacturers' instructions and dehydrated in increasing EtOH concentration gradient (70%, 95%, 100%) and mounted with Pertex (Histolab). Images of the developed emulsion and immunofluorescent stained sections were acquired with a Zeiss Observer Z.1 microscope (Carl Zeiss Microimaging GmbH, Jena, Germany) and processed using the ZEN software. An inverted lookup table was applied to the brightfield channel, resulting in white emulsion puncta instead of black for the final images. Quantification was performed with a standardized macro in ImageJ, as previously described [37].

### In vitro blood studies

Fresh blood from young and aged mice was collected directly from the heart with a heparinized needle under isoflurane anesthesia. The freshly collected blood from the same age groups was pooled to a total volume of 500 μL and was divided into aliquots of 10 × 50 μL. A high or low dose of ^125^I-labeled bispecific antibody (5 μL) was supplemented to the fresh heparinized blood aliquots at different time points: 120, 60, 15, 5 and 2 min. After the incubation time was finished for each sample and time point, samples were centrifuged at 10,000 *g* × 5 min at 4 °C and plasma was separated from the pellet. After the separation of plasma and pellet, radioactivity was measured by γ-counting (PerkinElmer). The procedure was done for each age group separately and repeated three times, with 2 young and 2 aged mice for each experiment (total n = 12).

### Western Blot

Capillary enriched brain pellets were isolated from frozen cerebrums, as described above ("Capillary depletion"). The capillary enriched pellets were washed with PBS, resuspended in 500 uL of Pierce RIPA lysis buffer (89,900, Thermo Fisher Scientific) with protease inhibitor cocktail (cOmplete mini tablets, Roche) and lysed on ice for 30 min in RT with gentle shaking. The samples were centrifuged at 14,000 *g* for 15 min at 4 °C. The supernatants were collected and total protein concentration was determined by BCA protein assay kit (23225, Thermo Fisher Scientific) according to the manufacturer's protocol. Protein samples were incubated in Bolt NuPAGE LDS sample buffer and Bolt sample reducing agent (Thermo Fisher Scientific) at 95 ℃ for 5 min. Next, 4 µg of protein per sample was loaded onto 4%–12% Bis–Tris Plus gels and run for 22 min at 200 V in Bolt MES-SDS running buffer. The separated protein samples were transferred to a nitrocellulose membrane using Bolt transfer buffer (Thermo Fisher Scientific) for 60 min at 20 V. The membranes were blocked with Odyssey TBS blocking solution (LI-COR Biosciences, Lincoln, USA) for 1 h and incubated overnight at 4 ℃ with primary antibodies; anti-transferrin (1:1000, 13-6899, Invitrogen) and anti-β actin (1:1000 ab8227, Abcam) as loading control. The following day, the membranes were washed with TBS-T (tris-buffered saline containing 0.1% Tween20, pH 7.4) before incubation for 1 h at RT with DyLightTM 800 goat anti-mouse (Thermo Fisher Scientific) and DyLightTM 680 goat anti-rabbit (Thermo Fisher Scientific) antibodies in 1:10 000 dilution. Finally, the membrane was washed with TBS-T and the fluorescence was measured using the LI-COR Odyssey-Sa reader (LI-COR Biosciences). The intensity of signal was visualized using LI-COR Image studio software.

### Statistical analyses

All data are presented as mean ± SD. The Shapiro–Wilk test was performed to test for normal distribution of the data. If all the data met the criteria for normality, unpaired t-tests or one-way analysis of variance (ANOVA) followed by Bonferroni's post hoc test were used, otherwise, Mann–Whitney U-test, or the Kruskal–Wallis test followed by Dunn's post hoc analysis was used. Pearson's correlation was applied to investigate the correlation between radioactivity (Bq) and brain concentration (%ID/gbrain) in the autoradiography images. Statistically significant p-values were defined as p < 0.05 (accounting for multiple comparisons), with annotations: *p < 0.05, **p < 0.01, ***p < 0.001 and ****p < 0.0001. Graphs and statistical analyses were made in GraphPad Prism version 9.5.0 (730) for Windows (GraphPad Software, San Diego, California USA).

## Results

Dose, or dose and body weight-corrected concentrations [%ID/g or %ID/g(bw)] (hereafter referred to as "concentrations") were used for comparison of the antibody distribution between low and high doses and between young and aged mice of different body weights.

The bispecific antibody ([^125^I]mAb3D6-scFv8D3) displayed sixfold and fourfold lower (at low and high dose respectively) concentrations in plasma compared with [^125^I]mAb3D6 (Fig. 1b) while bispecific antibody brain concentrations were 18-fold and 37-fold higher than those for the conventional antibody in young WT mice at 2 h post administration (Fig. 1c). These results verified bispecific antibody interaction with TfR1 both at the BBB and in blood and are in accordance previous observations for bispecific TfR1-Aβ antibodies of similar format [6].

### Whole blood, plasma and blood cell distribution

**Fig. 2:**
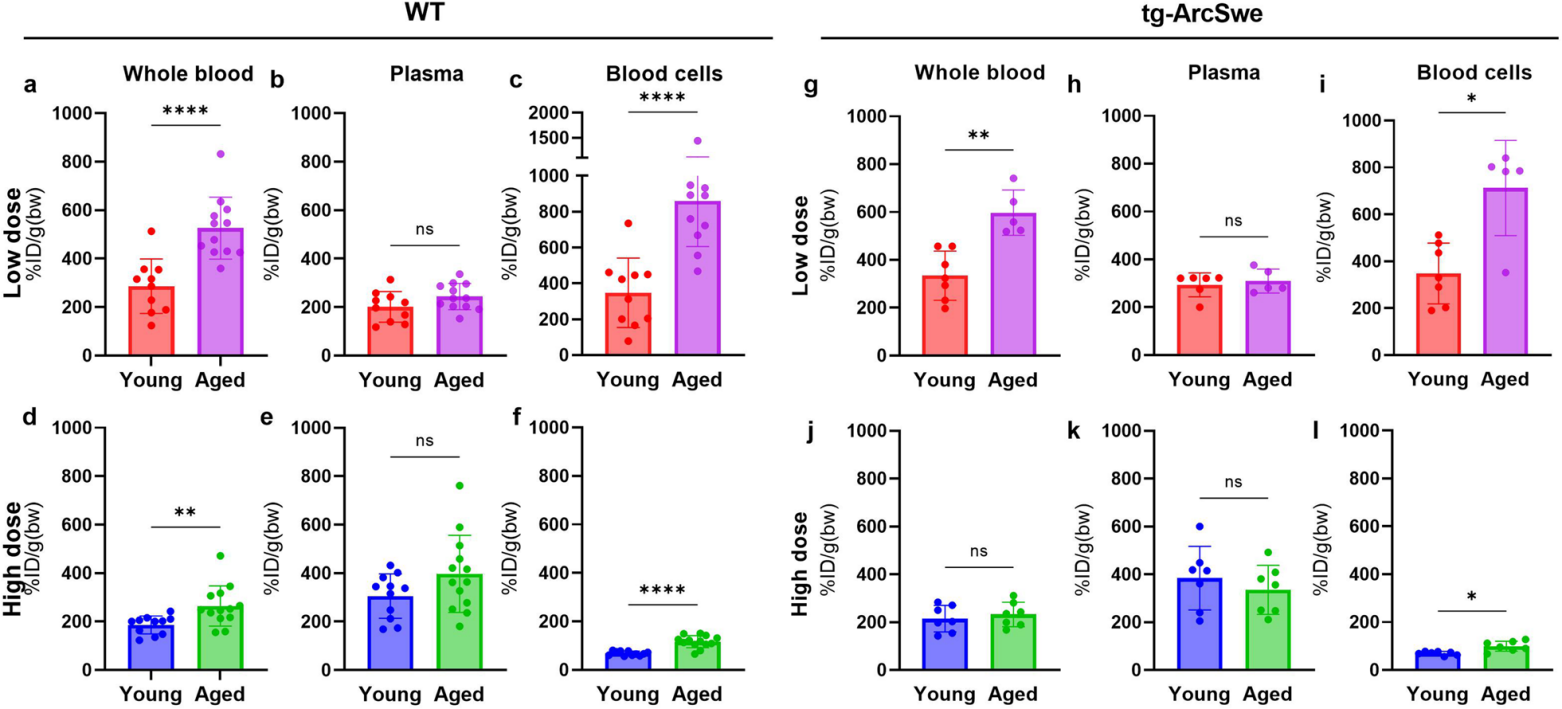
**a-l** Whole blood plasma and blood cell concentrations of [^125^I]mAb3D6-scFv8D3 in young and aged WT and tg-ArcSwe animals administered high and low dose of bispecific antibody (Mann–Whitney U-tests, significant p-value is defined as p < 0.05, *p < 0.05, **p < 0.01, ***p < 0.001 and ****p < 0.0001)

The whole blood concentration of the bispecific antibody was generally higher in aged compared with young mice at 2 h after injection (Fig. 2 a, d, g). The plasma concentrations of [^125^I]mAb3D6-scFv8D3 did not differ significantly between young and aged mice (Fig. 2 b, e, h, k). However, aged mice had significantly higher concentrations of the bispecific antibody in the blood cell fraction compared with the young mice, regardless of genotype and dose (Fig. 2c, f, i, l).

As expected, the low dose groups showed considerably higher concentrations of bispecific antibody in the blood cell fractions compared with the high dose groups (Fig. 2c, f, i, l). The bispecific antibody concentration in the blood cell fraction was especially elevated in the aged mice in the low dose group, compared with the high dose group (Fig. 2c, f, i, l).

Furthermore, there was no difference between tg-ArcSwe and WT mice in whole blood, plasma and blood cell [^125^I]mAb3D6-scFv8D3 concentrations when mice of the same age were compared. In line with what was observed in WT mice, aged tg-ArcSwe animals showed increased blood cell concentrations compared with young tg-ArcSwe, especially at the lower dose. However, for high dose, the aged mice did not differ significantly to young tg-ArcSwe mice in whole blood concentrations (Fig. 2j, k).

**Fig. 3:**
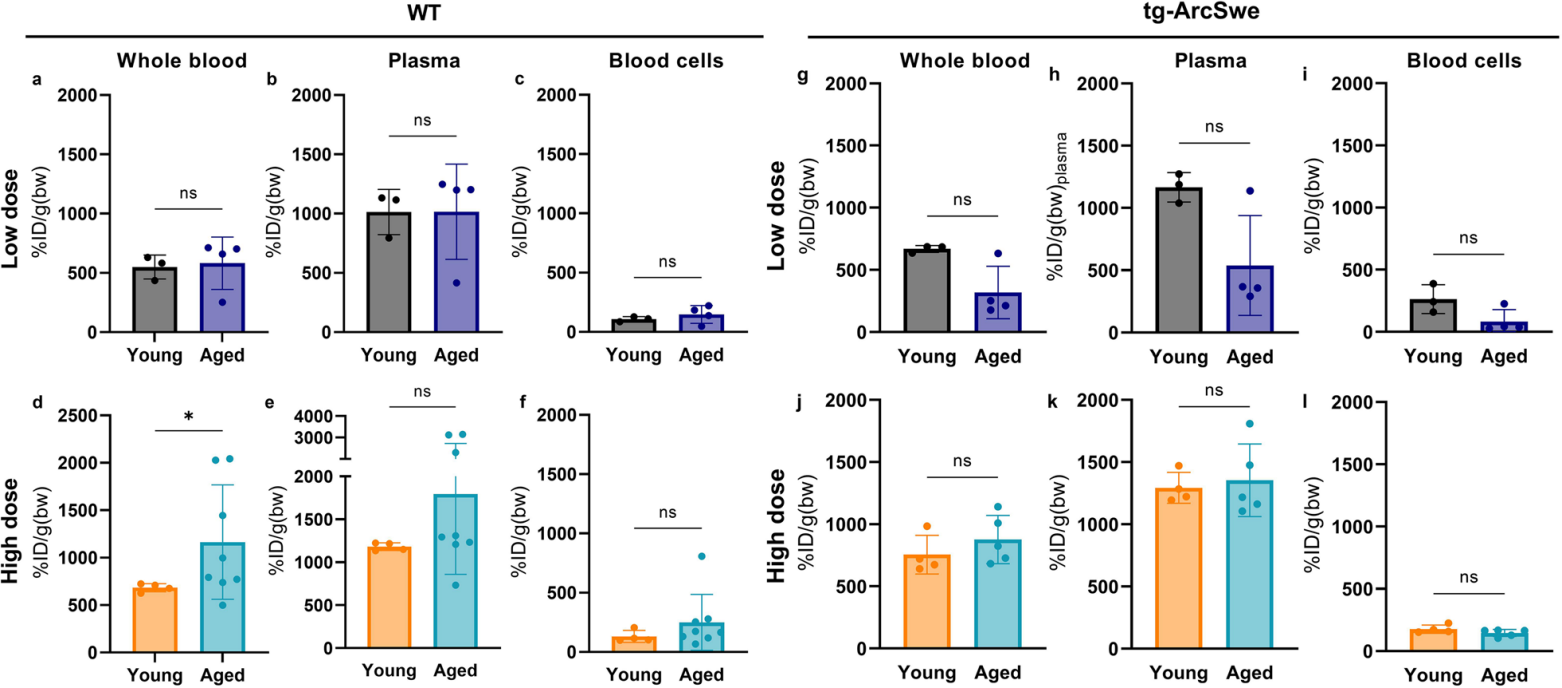
**a-l** Whole blood plasma and blood cell concentrations of [^125^I]mAb3D6 in young and aged WT and tg-ArcSwe animals administered high and low dose of conventional antibody (Mann–Whitney u-tests, significant p-value is defined as p < 0.05, *p < 0.05, **p < 0.01, ***p < 0.001 and ****p < 0.0001)

The conventional anti-Aβ antibody, [^125^I]mAb3D6, showed higher plasma levels compared with the bispecific antibody, especially at high dosing (Fig. 3 a, c, e, g). There were no significant differences in whole blood, plasma or the blood cell [^125^I]mAb3D6 concentration between ages, except that at high dose, the aged WT mice displayed somewhat higher blood concentrations than young mice (Fig. 3).

**Fig. 4:**
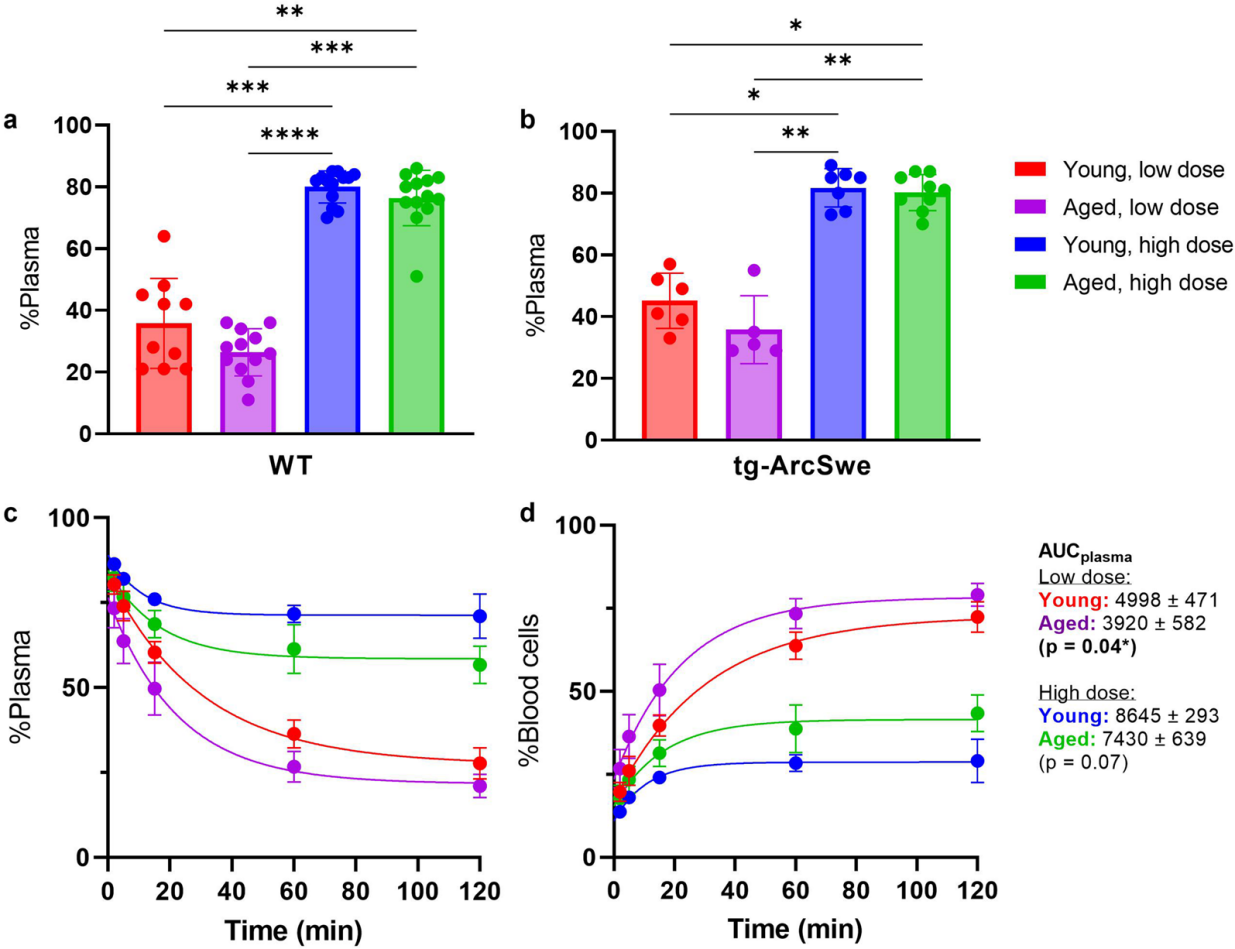
Percentage distribution to the plasma (%Plasma) vs blood cell fraction of [^125^I]mAb3D6-scFv8D3 in **a** WT and **b** tg-ArcSwe mice. Significant differences between low and high doses of [^125^I]mAb3D6-scFv8D3, as analysed with Kruskal–Wallis followed by Dunn's post-hoc analysis for multiple comparisons **c-d** Percentage in plasma and blood cell fractions over time after in vitro spiking with [^125^I]mAb3D6-scFv8D3 in blood from young and aged mice, at high and low dosing. AUC_plasma_ was significantly higher in the blood from young mice, compared with aged, at high dosing (p = 0.04), but did not reach statistical significance for low dose (p = 0.07) when analysed by pairwise one-way analysis of variance with Bonferroni correction. Significant p-value is defined as p < 0.05, *p < 0.05, **p < 0.01, ***p < 0.001 and ****p < 0.0001)

The percentage of [^125^I]mAb3D6-scFv8D3 measured in the plasma fraction at 2 h after injection was significantly higher in the high dosed groups, both for WT and tg-ArcSwe mice regardless of age or genotype (Fig. 4a, b). The young mice had a smaller plasma volume than the aged mice, while blood cell volumes were similar (Additional file 1: Fig. S1a, b). For low dosed animals, 34% ± 13% of the bispecific antibody was found in the plasma fraction, thus the majority of the bispecific antibody was residing in the blood cell fraction (Additional file 1: Fig S1c). Animals that received a high dose of the bispecific antibody had 79% ± 7% antibody in the plasma fraction, which was similar to the conventional antibody [^125^I]mAb3D6 (85% ± 9% and 89% ± 5%, low and high dose respectively) (Additional file 1: Fig. S1c).

To further study the time aspects of antibody association to the blood cell fraction, whole blood was spiked with the radiolabeled bispecific antibody. A faster association to blood cells, i.e. a faster decline in plasma concentration over time, was observed after spiking blood with a low dose of [^125^I]mAb3D6-scFv8D3 compared to when a high dose was used (Fig. 4c, d). A higher association of bispecific antibody to the blood cell fraction was seen in blood sampled from aged mice compared to when the blood had been obtained from young mice (Fig. 4c, d). AUC_plasma_ was significantly higher in the blood from young mice, compared with aged mice, at high dosing (p = 0.04), but did not reach statistical significance for the low dose (p = 0.07). The bispecific antibody concentrations in plasma and blood cell fractions appeared to reach an equilibrium after around 60 min post addition of [^125^I]mAb3D6-scFv8D3 (Fig. 4 c, d).

Some accumulation in the spleen (an organ associated with high TfR1 levels) was seen in the animals injected with the bispecific antibody, but not in mice injected with the regular antibody indicating peripheral TfR1 binding beyond blood (Additional file 1: Fig S2b, Fig. S3a, b). Mice injected with a low dose of bispecific antibody showed significantly higher concentration in the spleen compared with animals that received a high dose, both in WT and in tg-ArcSwe mice (Additional file 1: Fig S3a, b).

### Brain delivery 2 h post-injection

**Fig. 5:**
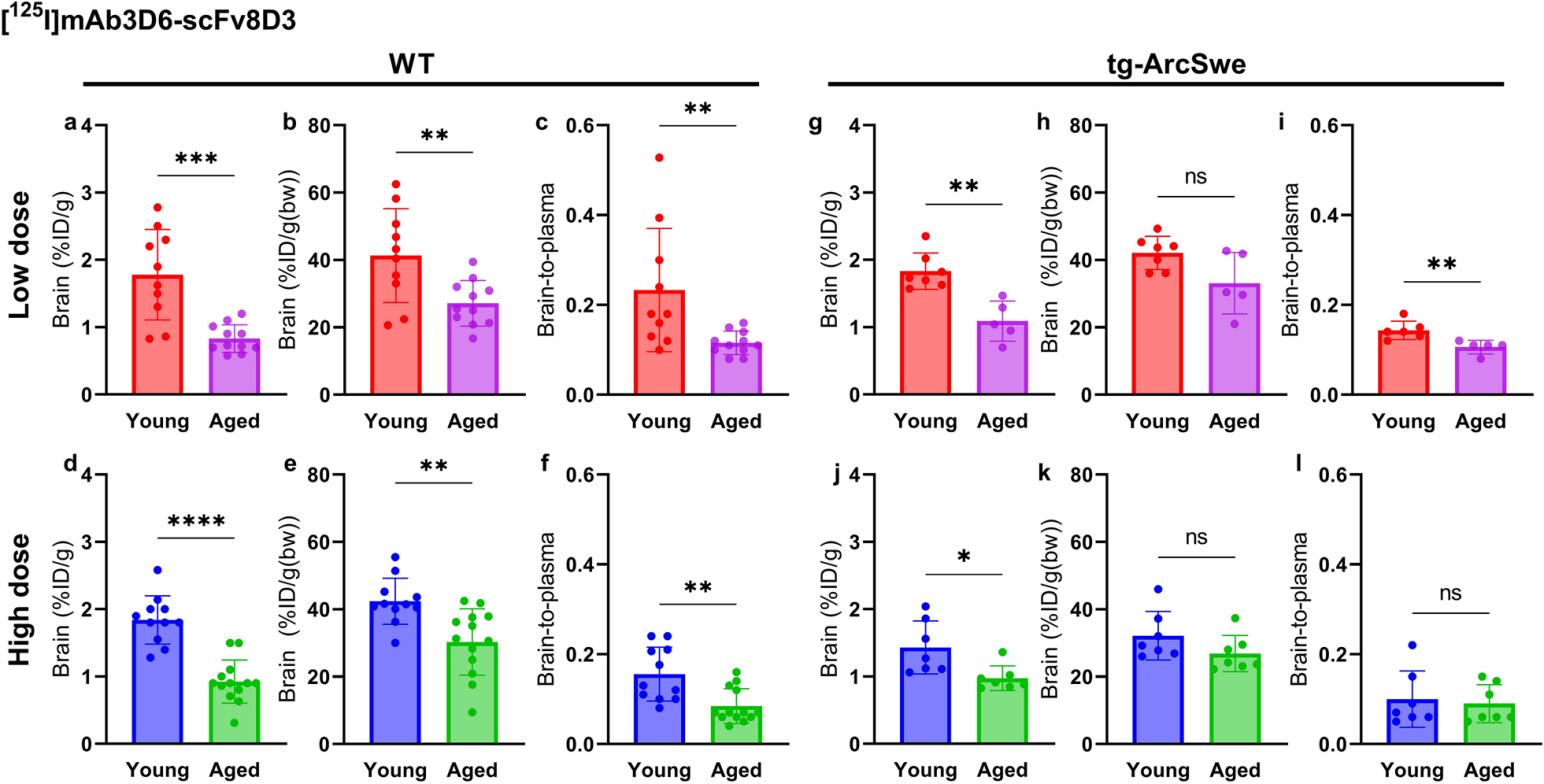
**a–l** Brain concentration (%ID/g brain), %ID/g/bw and brain-to-plasma ratios for WT and tg-ArcSwe mice, administered bispecific [^125^I]mAb3D6-scfv8D3 at high and low dose. To test significance in the results between young and aged mice, unpaired t-tests were used for brain concentrations; %ID/g_Brain_ or %ID/g(bw)_Brain_ and Mann–Whitney U-tests were used for brain-to-plasma ratios, due to non-normality. Significant p-value is defined as p < 0.05, *p < 0.05, **p < 0.01, ***p < 0.001 and ****p < 0.0001)

The young WT mice showed higher [^125^I]mAb3D6-scFv8D3 brain concentrations (%ID/g) at 2 h after injection compared with the aged WT mice. This was true both after administering a low and a high dose of the bispecific antibody. The difference was still significant when adjusting for body weight between young and aged animals (%ID/g(bw)), and in the brain-to-plasma ratios (Fig. 5a–f). In line with the results in WT mice, there was also a difference in brain concentrations of [^125^I]mAb3D6-scFv8D3 between young and aged tg-ArcSwe mice. However, this difference was not significant when the body weight corrected concentration was used, (p = 0.05 low dose and p = 0.14 for high dose), nor in brain-to-plasma concentration ratios for the high dosed group (Fig. 5 g–l). Further, there was no significant difference between WT and tg-ArcSwe of the same age and dose group, in brain concentrations (%ID/g(bw)) of [^125^I]mAb3D6-scFv8D3 (Additional file 1: Fig. S4).

**Fig. 6:**
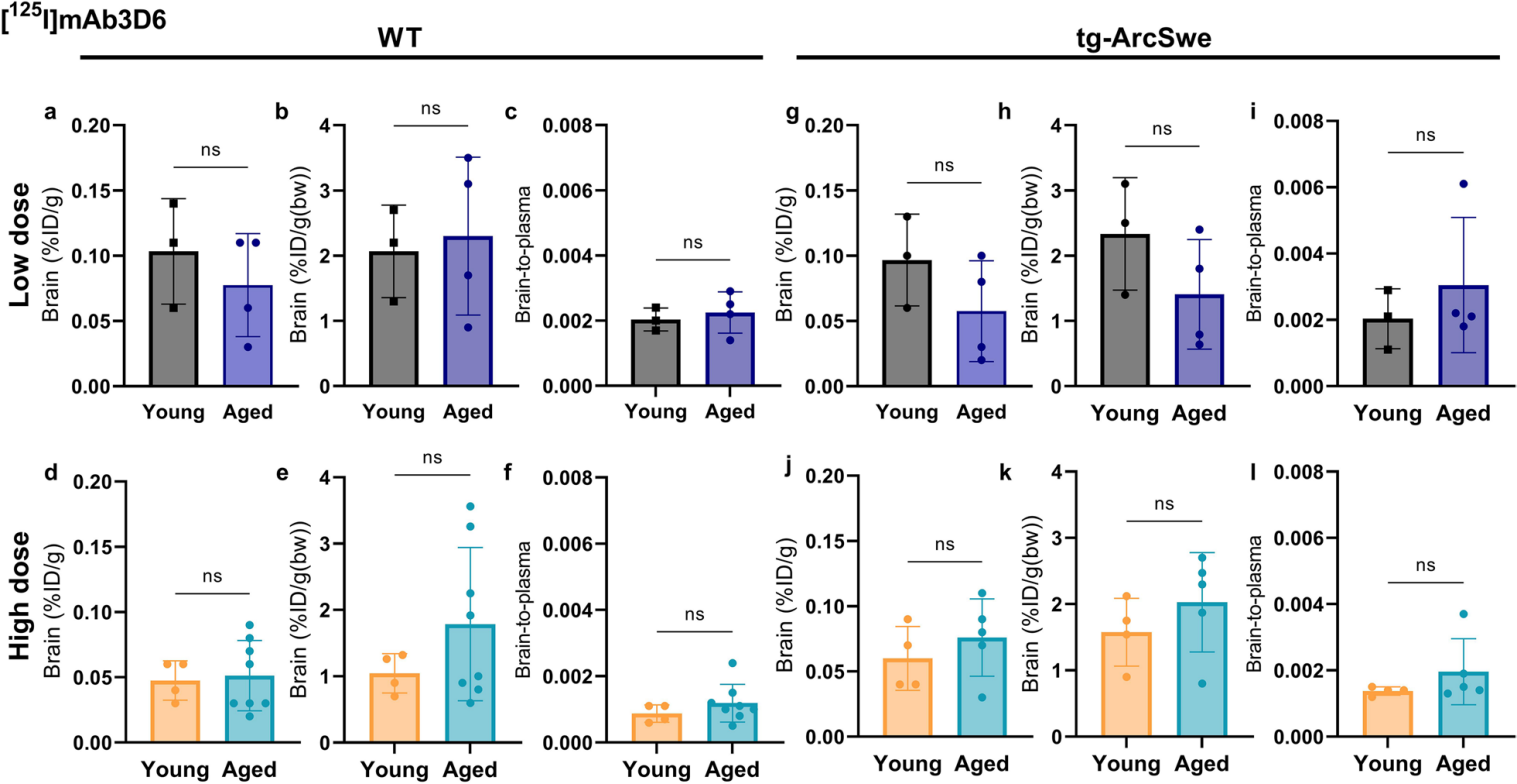
**a–l** Brain concentration (%ID/g brain), %ID/g/bw and brain-to-plasma ratios for WT and tg-ArcSwe mice, administered [^125^I]mAb3D6 at high or low dose. To test significance in the results between young and aged mice, unpaired t-tests were used for brain concentrations; %ID/g_Brain_ or %ID/g(bw)_Brain_ and Mann–Whitney *U*-tests were used for brain-to-plasma ratios, due to non-normality. Significant p-value is defined as p < 0.05, *p < 0.05, **p < 0.01, ***p < 0.001 and ****p < 0.0001)

For [^125^I]mAb3D6, no significant differences in brain concentrations and brain-to-plasma concentration ratios could be detected between young and aged mice, nor between WT and tg-ArcSwe animals (Fig. 6). This indicated that brain delivery of [^125^I]mAb3D6 was not dependent on age, dose nor pathology. Brain-to-plasma concentration ratios revealed a 100-fold difference between [^125^I]mAb3D6 and [^125^I]mAb3D6-scFv8D3 (Additional file 1: Fig. S5).

### Therapeutic dose in young WT mice

Brain and blood distribution was further investigated in a group of young WT mice administered with a therapeutic dose of 10 mg/kg of bispecific mAb3D6-scFv8D3, i.e. a tenfold higher dose than the high dose in the previous experiments.

**Fig. 7:**
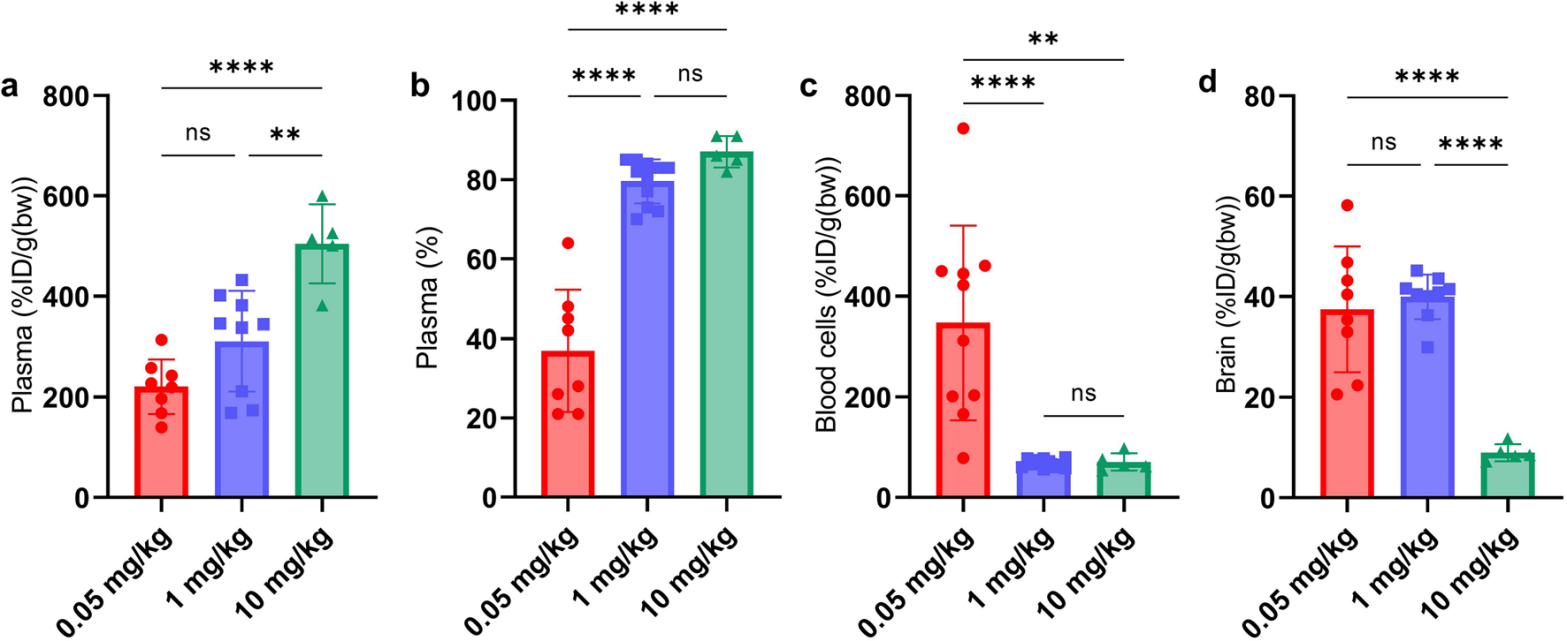
**a** Plasma (%ID/g(bw)) concentrations, **b** percentage of bispecific antibody in the plasma fraction (%) **c** blood cell pellet (%ID/g(bw)) concentrations and **d** brain concentrations (%ID/g(bw)) after 0.05, 1 or 10 mg/kg-administration of [^125^I]mAb3D6-scFv8D3 in young WT mice. Significant differences were analysed by one-way ANOVA with Bonferroni's posthoc test. Significant p-value is defined as p < 0.05, *p < 0.05, **p < 0.01, ***p < 0.001 and ****p < 0.0001)

Plasma concentrations of [^125^I]mAb3D6-scFv8D3 were elevated compared with dosing at 1 mg/kg or 0.05 mg/kg (Fig. 7a). The percentage of antibody in plasma was 87% ± 3.4%, i.e. not statistically different from the 1 mg/kg dose (Fig. 7b). Blood cell pellet concentrations after the 10 mg/kg dosing was also similar to what was observed for the 1 mg/kg dose (Fig. 7c). However, the dose normalised brain concentrations, %ID/g(bw), were drastically decreased compared with the 0.05 and 1 mg/kg doses, likely attributed to saturation of TfR1 at the BBB (Fig. 7d), in accordance with previous observations [49].

### Ex vivo autoradiography

**Fig. 8:**
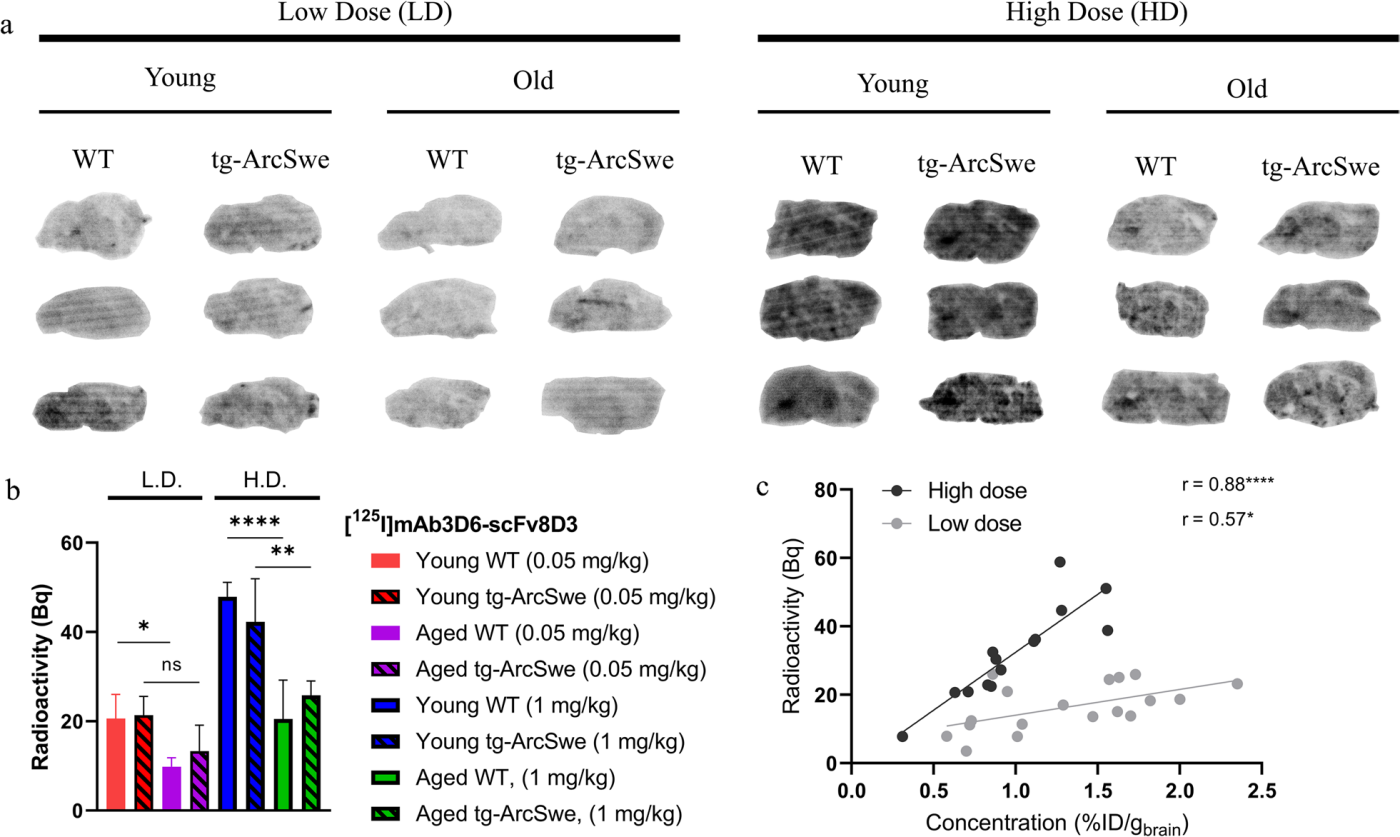
**a** Ex vivo autoradiography of representative brain sections (NB not corrected for injected dose or weight of animal) **b** Quantification of brain section radioactivity of the whole brain area (ROI). Pairwise comparisons between young and aged mice of each genotype were done using one-way analysis of variance, with Bonferroni correction **c** Correlation between radioactivity (Bq) and brain concentration (%ID/g brain), statistical analysis by Pearson's correlation test (low dose: r = 0.57*, p = 0.01, high dose: r = 0.88****, p < 0.0001). Significant p-value is defined as p < 0.05, *p < 0.05, **p < 0.01, ***p < 0.001 and ****p < 0.0001)

Ex vivo autoradiography illustrated the 2 h post-injection brain radioactivity derived from [^125^I]mAb3D6-scFv8D3. The highest total radioactivity was observed in the young mice that had received the high dose (1 mg/kg) (Fig. 8a), with significantly higher radioactivity compared to the aged groups (Fig. 8b). Within the low dose groups, young animals also displayed higher radioactivity than aged animals. Moreover, radioactivity in sections prepared from WT and tg-ArcSwe mice did not differ, for either low or high dose (Fig. 8a).

### Dose-dependent parenchymal delivery of bispecific antibody

**Fig. 9:**
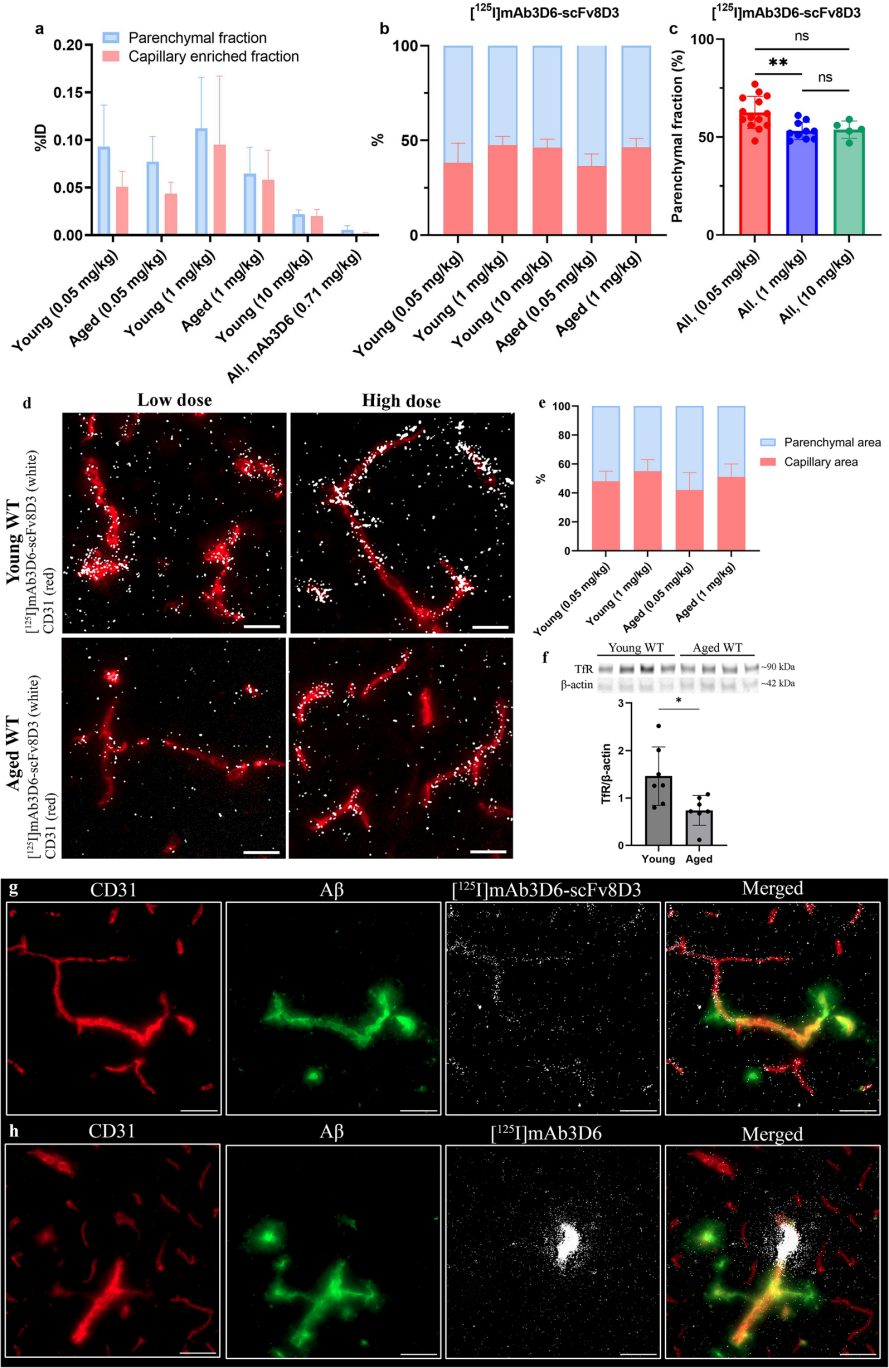
**a** Capillary depletion displayed as % of injected radioactivity (%ID) in parenchymal or capillary enriched fractions **b** [^125^I]mAb3D6-scFv8D3-distribution between parenchymal and capillary-enriched fraction (%) for different age and dose groups **c** parenchymal distribution (%) of [^125^I]mAb3D6-scFv8D3, pooled by dose group. Significant differences were tested with one-way analysis of variance with Bonferroni correction for multiple comparisons. For capillary depletion: young, high n = 4, young low n = 7, aged high n = 6, aged low n = 7, young 10 mg/kg n = 5, all [^125^I]mAb3D6 n = 13. **d** Representative images of nuclear track emulsion (NTE), with [^125^I]mAb3D6-scFv8D3 shown as white puncta, and vascular marker CD31 in red. Scale bar = 20 µm. **e** Quantification of percentage in parenchymal or capillary ROIs, n = 4 for all groups. **f** Representative western blot of TfR1 levels in capillary-enriched brain pellets from young and aged WT mice and quantification relative to loading control protein (β-actin). Whole membrane can be found in Fig.S8 (Additional file 1). Statistical analysis by unpaired t-test. Significant p-value is defined as p < 0.05, *p < 0.05, **p < 0.01, ***p < 0.001 and ****p < 0.0001). **g**, **h** Nuclear track emulsion with flourecent stainings of vasculature (CD31-red) and Aβ40 (green) of [^125^I]mAb3D6-scFv8D3-injected or [^125^I]mAb3D6-injected aged tg-ArcSwe mice. Scale bar = 50 µm

Capillary depletion of brain homogenates was used to separate antibody distribution into a parenchymal and a capillary enriched fraction. The dose corrected concentrations (%ID) showed that the bispecific antibody, [^125^I]mAb3D6-scFv8D3, distributed to both fractions at low and high dosing. However, the therapeutic dose of 10 mg/kg resulted in a fourfold lower %ID in the fractions compared with 1 mg/kg or 0.05 mg/kg doses (Fig. 9a). The overall %ID of conventional antibody [^125^I]mAb3D6 in the parenchymal fraction was 15-fold lower than for the bispecific antibody, reflecting the low brain uptake of [^125^I]mAb3D6 (Fig. 9a).

The relative distribution (%) of the bispecific antibody between parenchymal and capillary enriched fraction did not differ between WT and tg-ArcSwe mice, nor between young and aged animals (Additional file 1: Fig. S7 a, b). However, after low dosing of the bispecific antibody (0.05 mg/kg), there was a trend towards higher relative parenchymal distribution (%) compared to high dosing (1 mg/kg), both for young and aged mice (Fig. 9b,c).

Nuclear track emulsion was used to visualize of [^125^I]mAb3D6-scFv8D3 in brain capillaries. The young animals displayed a more concentrated [^125^I]mAb3D6-scFv8D3-derived signal in capillaries (CD31 positive areas) than aged animals. The difference was clearer after a high dose then after a low dose (Fig. 9d). When the signal was quantified in the parenchymal and capillary areas, there was a trend, in line with the capillary depletion, towards a higher relative signal in the parenchymal area after a low dose compared to a high dose (Fig. 9e). Western blot analysis of total TfR1 levels in isolated capillary enriched brain pellets, from naïve mice, indicated higher levels of TfR1 protein in young compared with aged mice (Fig. 9 f and Additional File 1: S8a, b).

NTE in aged tg-ArcSwe animals further revealed diffrences in the distribution between the two antibodies, [^125^I]mAb3D6-scFv8D3 and [^125^I]mAb3D6 (Fig. 9 g, h). The bispecific antibody displayed some localization in proximity to vascular pathology, but the signal was generally stronger in vessels without Aβ pathology. This is in high contrast to [^125^I]mAb3D6 that mainly co-localized with vascular pathology, but only at few “hot spots” in the brain. Overview images of the distribution of the two antibodies in different brain regions in aged WT and tg-ArcSwe mice (at a high dose) can be found in Additional file 2.

Although the overall [^125^I]mAb3D6 concentration in capillary depletion was very low (Fig. 9a), the distribution to capillary enriched pellet was significantly increased in the aged tg-ArcSwe mice compared to both young and aged WT mice. This could further support a possible retention of [^125^I]mAb3D6 at vascular pathology, present in the aged tg-ArcSwe mice (Additional file 1: Fig S6).

## Discussion

In this paper, we have explored the blood and brain distribution of a bispecific antibody, mAb3D6-scFv8D3, and the conventional anti-Aβ antibody mAb3D6 at 2 h after i.v. administration. The study was carried out in young and aged WT and tg-ArcSwe mice at different antibody doses. The main finding was that aged mice exhibited a decreased brain uptake of the bispecific antibody mAb3D6-scFv8D3 compared with young mice, while brain distribution of mAb3D6 was not influenced by age. The lower bispecific antibody brain concentrations in aged mice were observed both after a tracer dose of 0.05 mg/kg and at dose of 1 mg/kg. Although still apparent, the difference between young and aged mice was less prominent in tg-ArcSwe than in WT mice. This may potentially be a consequence of the abundant brain and vascular Aβ pathology present in aged tg-ArcSwe mice, or presence of Aβ in blood, that could influence brain delivery and antibody brain retention.

Further, depending on dose, [^125^I]mAb3D6-scFv8D3 displayed 18- to 37-fold higher brain concentrations than [^125^I]mAb3D6 at 2 h. This was anticipated as [^125^I]mAb3D6 brain distribution is likely to rely mainly on slow, unspecific transport [50], while [^125^I]mAb3D6-scFv8D3 binds TfR1 at the BBB, and thus, enters the brain via transcytosis across the capillary cells fairly fast [5]. The difference in brain distribution in different brain regions between the two antibodies was also clearly demonstrated by NTE imaging. Strong vascular signals for [^125^I]mAb3D6-scFv8D3 was shown at this time point, and a brain-wide distribution, mainly at Aβ-negative vessels. This indicates that [^125^I]mAb3D6-scFv8D3 brain uptake is mainly driven by TfR-binding at this time point (2 h p.i.). In contrast, [^125^I]mAb3D6 had high distribution at the choroid plexus, pial surfaces, and locally at some areas with vascular Aβ pathology in the aged tg-ArcSwe mice (Additional file 2).

Although debated, aging has generally been linked to increased permeability of larger molecules at the BBB due to age-induced BBB leakiness [33, 51–54]. Yang et al. attributed this to a shift in transcytosis from ligand-specific receptor-mediated to non-specific caveolar transcytosis that occurs during aging, resulting in increased non-specific uptake of IgGs [33]. However, in the present study, we found no significant age-dependent difference in [^125^I]mAb3D6 brain uptake, indicating that mAb3D6 brain delivery was not affected by an overall age-dependent or Aβ-pathology caused leakiness of the BBB. One explanation could be that the time-point chosen here, 2 h, does not reflect the maximum concentrations of [^125^I]mAb3D6, since it is known that conventional antibodies have a slower brain uptake, as shown in our previous work (Gustavsson et al. 2020), with maximum brain concentrations occurring around a few days after injection [30]. Further, our previous findings using [^125^I]mAb3D6 and differently sized dextrans showed a low impact on antibody [^125^I]mAb3D6 permeability in old AD mice 3 days after injection [55]. Therefore we believe that the dramatic effects on brain concentrations of [^125^I]mAb3D6-scFv8D3 seen between young and aged mice in the present study were mainly related to TfR1 binding, either at the BBB or in the periphery.

Although our data on the conventional IgG differ from Yang et al.’s observations, their report on reduced specific transcytosis at the BBB in aged mice are in line with the results for the bispecific antibody [33]. They showed that TfR expression, as quantified by mRNA levels and fluorescence imaging, was significantly decreased in 20–24 month old mice, compared with young 3 month mice [33]. In non-dosed animals, we observed higher TfR1 protein levels in brain capillaries isolated from young mice compared to those isolated from aged mice, which could explain the higher brain concentrations of [^125^I]mAb3D6-scFv8D3 in young than in aged mice. In line with this, a previous study reported a tendency towards lowered TfR1 levels in WT mice of 22 months compared with 8 months [32]. Contrary to this, Bourassa et al. reported no differences in TfR1 levels between 12-month old and 18-month old mice, nor in brain uptake of the fluorescently labeled high affinity TfR1-antibody Ri7 after 1 min of in situ perfusion [31]. It should be noted that 12-month old mice may not be regarded as "young", and thus, a potential explanation to these somewhat conflicting observations is that TfR1 levels may decrease before the age of 12 months.

Further indications that senescence is related to impaired transcytosis of scFv8D3-transported antibodies come from in vitro BBB cell models, where a lowered transcytosis efficiency of bispecific TfR1-binding antibody was observed at higher cell passages [56]. Other physiological factors that could impede TfR1-mediated transport at the BBB in old age include; a thicker glycocalyx at the luminal side of the BCECs (decreasing the availability of TfR1) or a thicker base membrane (inhibiting the transport to parenchyma) [57–59]. The literature, as well as our results, suggest that TfR1-antibody brain concentrations are not widely affected in AD-mouse models compared with age-matched WT mice [30, 31].

To further study the brain delivery, we measured antibody concentrations in capillary and parenchymal compartments by capillary depletion and NTE. The relative parenchymal delivery, i.e. the efficiency of [^125^I]mAb3D6-scFv8D3 to cross the BBB, was not age- or genotype-dependent (although absolute concentrations for the young mice was higher in both of these compartments compared with the aged mice). There was a trend towards a relatively higher distribution to the parenchyma after a low dose of [^125^I]mAb3D6-scFv8D3, compared with a high dose. This is in line with previous observations for TfR1 antibodies showing that, at tracer doses, high affinity binders cross the BBB better while at therapeutic doses, low affinity binders transcytose better [11]. Interestingly, although the total levels measured in capillary fractions for the conventional antibody [^125^I]mAb3D6 was low, it was increased in aged tg-ArcSwe mice, supporting the notion that presence of Aβ pathology can increase vascular retention of this antibody [55, 58]. Interaction between [^125^I]mAb3D6 and Aβ in the vasculature was also revealed by NTE. Interestingly, for [^125^I]mAb3D6-scFv8D3, the vascular signal was stronger at vessels without Aβ pathology, indicating that the brain concentrations were not driven binding to vascular Aβ, but rather TfR1, at this time point.

Generally, brain concentrations are driven by peripheral concentrations, and thus, antibody concentrations in whole blood, plasma and blood cell pellet (after separation of plasma) was investigated further to explore the differences between young and aged mice. The plasma concentrations of [^125^I]mAb3D6-scFv8D3 were similar in young and aged mice in vivo at 2 h, but the aged mice displayed higher concentrations in blood cell pellet compared with young mice. This difference was especially evident at low dose, i.e. when binding to TfR1 was not saturated. In contrast to the in vivo blood samples, the in vitro blood binding to TfR1 could be determined before equilibrium between plasma and blood cell compartments was reached (around 60 min) and suggested that young mice had a higher AUCplasma, i.e. lower extent and slower distribution of [^125^I]mAb3D6-scFv8D3 to blood cells, than aged mice. Taken together, these data indicated that a larger fraction of the bispecific antibody may be available in plasma for BBB transcytosis in young than in aged mice. Age has been associated with increased number of reticulocytes in mice [60]. A report in similar ages as those used in this present study, showed that 24 months old mice had 50% more reticulocytes than 2 months old animals [61]. This could indicate that TfR1 levels in blood (and, and as a result, sTfR1 levels in plasma [62, 63]) are increased in aged mice, negatively impacting the distribution and availability of [^125^I]mAb3D6-scFv8D3 for binding at the BBB.

The elimination from blood was faster for [^125^I]mAb3D6-scFv8D3 than for [^125^I]mAb3D6, consistent with TfR1 mediated clearance [64]. This resulted in a lower distribution of the bispecific antibody to plasma compared with [^125^I]mAb3D6, especially at low dose, when [^125^I]mAb3D6-scFv8D3 likely was sequestered by TfR1 expressed by blood cells. When the bispecific antibody was administered at the low dose of 0.05 mg/kg only 34% of the antibody was found in the plasma, while 66% was associated with the blood cell pellet. The numbers were nearly reversed, and similar to what was observed for the conventional mAb3D6 antibody, after a dose of 1 mg/kg with 79% found in plasma and 21% in blood cell pellet. Furthermore, after a therapeutic dose of 10 mg/kg [^125^I]mAb3D6-scFv8D3, plasma concentrations were further increased, indicating that at high doses, peripheral TfR-binding is likely to be of less importance for the availability of the bispecific antibody in plasma. This also indicates that TfR1-binders may exhibit an 'optimal dosing window' in which TfR1-binding in blood, but not at the BBB, is saturated. For [^125^I]mAb3D6-scFv8D3, this optimal dose may be around 1 mg/kg, but it is likely that the affinity towards TfR1 will influence this window and the optimal dose may therefore differ between different TfR1 binders. A similar bell-shaped relationship between affinity and brain uptake has been suggested, where low TfR-affinity leads to higher systemic exposures, but impeded BBB binding, and high affinity TfR-binders are more rapidly cleared from the blood, and are subjected to TfR-mediated degradation, which also can inhibit the brain delivery [64, 65]. The choice of dose will also differ depending on the application of the bispecific antibodies. For therapeutic purposes, it is important to maximise the fraction of administered antibodies that will be delivered to the brain and to enable repeated injections, i.e. by avoiding down-regulation of TfR1 or toxicity [11, 34]. Thus, it has been suggested that monovalent binding and moderate affinity towards the TfR1 is desired for therapeutic use [18, 66]. However, for diagnostic use, such as when used as PET-radioligands, bispecific antibodies can be dosed at much lower doses, and high affinity may even be beneficial for rapid brain entry [6, 9, 67, 68].

The present study was carried out using one single time point (2 h post injection) for measurement of brain concentrations and thus only provided a "snap-shot" of brain delivery. The early time point was chosen based on the assumption that TfR-binding would be the main driver of brain delivery at this time, while Aβ-binding is important at later time points [5, 6, 9]. Although a limitation, the rationale for this experimental design was also to reduce the number of animals needed to study the effects of the other factors, i.e. genotype, dose and age. However, studying brain concentrations over time could potentially reveal further differences between the ages. For example, brain uptake and clearance likely occurs simultaneously, and both early uptake and late elimination from the brain could differ between the ages. Another limitation in the study is that the bispecific antibody may bind bivalently, with its two inherent TfR-binders, at least at higher doses [69]. The binding mode may be influenced by the dose with a more monovalent type of binding at lower doses, but more bivalent at higher doses. It would therefore be of value to further study the influence of factors discussed in this paper for a strictly monovalent antibody.

In conclusion, we have described age-dependent differences in blood and brain concentrations at an early time point, 2 h, after administration of a bispecific antibody targeted towards TfR1 and Aβ. These differences were not observed for a conventional Aβ antibody; thus, we suggest that differences were TfR1-related. The observed decrease in brain antibody concentrations in aged mice compared to young mice was likely a consequence of decreased TfR1-mediated BBB delivery in aged mice, potentially in combination with increased blood cell binding that reduced the amount of available antibody in plasma. Brain delivery did not differ between same-age AD and WT mice, indicating that Aβ-pathology did not influence the early brain distribution of the bispecific antibody. Low doses of bispecific antibody were associated with increased blood cell binding, but also with increased relative parenchymal delivery, whereas therapeutic doses showed increased availability in plasma, but at the same time lower brain distribution likely as a consequence of TfR1 saturation at the BBB. Taken together, animal age and bispecific antibody dose were important factors influencing brain delivery, while genotype was not.

## Supplementary Information

**Additional file 1: Figure S1**. **a** Lower plasma volume in young mice compared with aged **b** No difference in blood cell volumes between young and aged mice **c** Percentage in plasma of [^125^I]mAb3D6-scFv8D3 and [^125^I]mAb3D6 in young and aged mice at low or high doses **Figure S2. a** Weight of spleen in young and aged mice **b** %ID/spleen of bispecific or regular antibody at high and low doses. **Figure S3. a** %ID/spleen of [^125^I]mAb3D6-scFv8D3 in young and aged, and low and high doses in **a** WT and **b** tg-ArcSwe mice. **Figure S4.** %ID/g/bw brain of [^125^I]mAb3D6-scFv8D3. No difference between WT and tg-ArcSwe in brain uptake after 2 h. **Figure S5.** Brain-to-plasma ratios for [^125^I]mAb3D6-scFv8D3 or [^125^I]mAb3D6 -injected mice at low or high dosing. **Figure S6. **Capillary depletion for [^125^I]mAb3D6-administered mice at high dose, expressed as percentage in parenchymalor capillary enrichedfractions. **Figure S7. a **Capillary depletion for all low [^125^I]mAb3D6-scFv8D3-dosed mice, and **b **all high dosed mice, expressed as percentage in parenchymalor capillary enrichedfractions. **Figure S8.** Full membrane of capillary enriched brain pellets detected with anti-TfR1 antibody for TfR1, and anti-β-actin, β-actinas loading control.

**Additional file 2: Figure 1**. Nuclear track emulsion at different brain regions in [125I]mAb3D6-scFv8D3 or [125I]mAb3D6-injected mice.

## Data Availability

The datasets used and/or analysed during the current study are available from the corresponding author on reasonable request.

## Abbreviations

Aβ: Amyloid beta
AD: Alzheimer's disease
AUC: Area under the curve
BBB: Blood–brain barrier
CSF: Cerebrospinal fluid
IgG: Immunoglobulin G
mAb: Monoclonal antibody NTE: nuclear track emulsion
RMT: Receptor mediated transcytosis
TfR1: Transferrin receptor 1
tg: Transgenic
scFv: Single chain variable fragment
sTfR: Soluble Transferrin Receptor
PET: Positron Emission Tomography
WT: Wild type
