# Supplementary material for: Age, dose, and binding to TfR on blood cells influence brain delivery of a TfR-transported antibody
Source: Fluids Barriers CNS. 2023 May 11;20:34. doi: 10.1186/s12987-023-00435-2 (PMC10173660; doi:10.1186/s12987-023-00435-2)
Supplement: Supplementary file 1 — Additional file 1: Figure S1. a Lower plasma volume in young mice compared with aged b No difference in blood cell volumes between young and aged mice c Percentage in plasma of [125I]mAb3D6-scFv8D3 and [125I]mAb3D6 in young and aged mice at low or high doses Figure S2. a Weight of spleen in young and aged mice b %ID/spleen of bispecific or regular antibody at high and low doses. Figure S3. a %ID/spleen of [125I]mAb3D6-scFv8D3 in young and aged, and low and high doses in a WT and b tg-ArcSwe mice. Figure S4. %ID/g/bw brain of [125I]mAb3D6-scFv8D3. No difference between WT and tg-ArcSwe in brain uptake after 2 h. Figure S5. Brain-to-plasma ratios for [125I]mAb3D6-scFv8D3 or [125I]mAb3D6 -injected mice at low or high dosing. Figure S6. Capillary depletion for [125I]mAb3D6-administered mice at high dose, expressed as percentage in parenchymalor capillary enrichedfractions. Figure S7. a Capillary depletion for all low [125I]mAb3D6-scFv8D3-dosed mice, and b all high dosed mice, expressed as percentage in parenchymalor capillary enrichedfractions. Figure S8. Full membrane of capillary enriched brain pellets detected with anti-TfR1 antibody for TfR1, and anti-β-actin, β-actinas loading control. [file 12987_2023_435_MOESM1_ESM.docx]

**Additional file**

**Blood**

**Fig S1**. **a** Lower plasma volume in young mice (unpaired t-test) compared with aged **b** No difference in blood cell volumes between young and aged mice (unpaired t-test) **c** Percentage in plasma of [^125^I]mAb3D6-scFv8D3 and [^125^I]mAb3D6 in young and aged mice at low or high doses, analyzed by one-way ANOVA with Bonferroni correction for multiple comparisons.


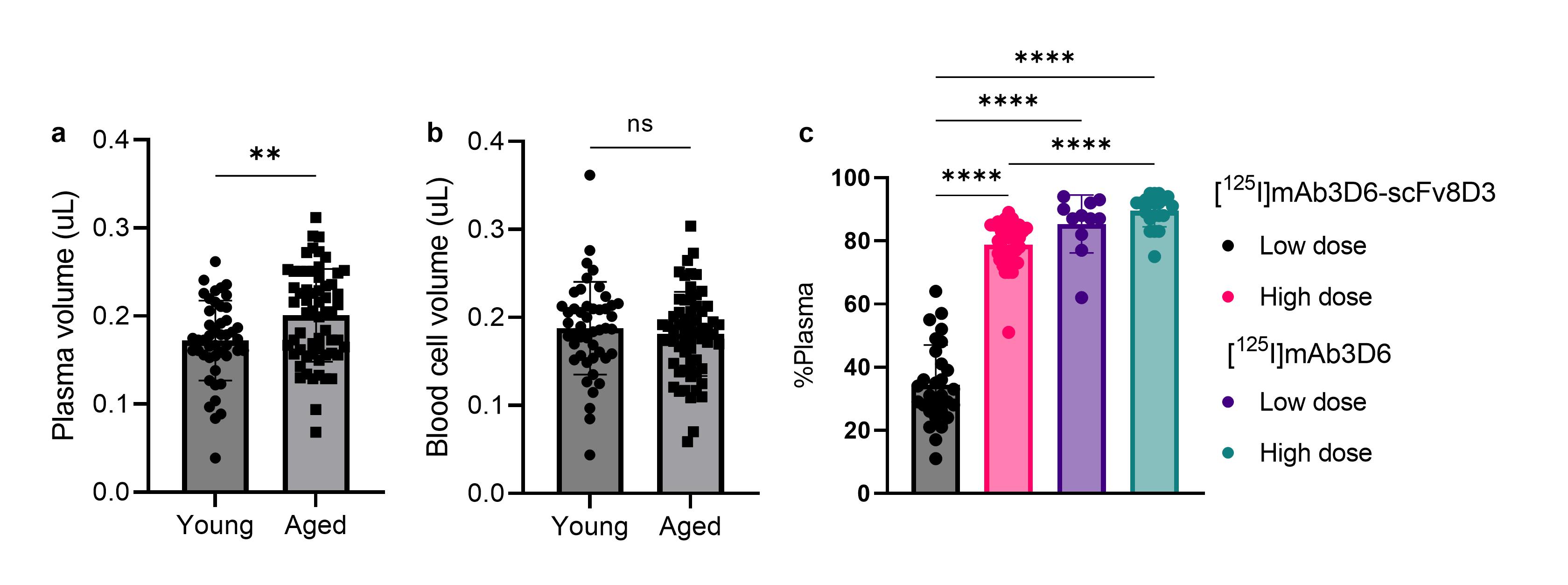


**Spleen**


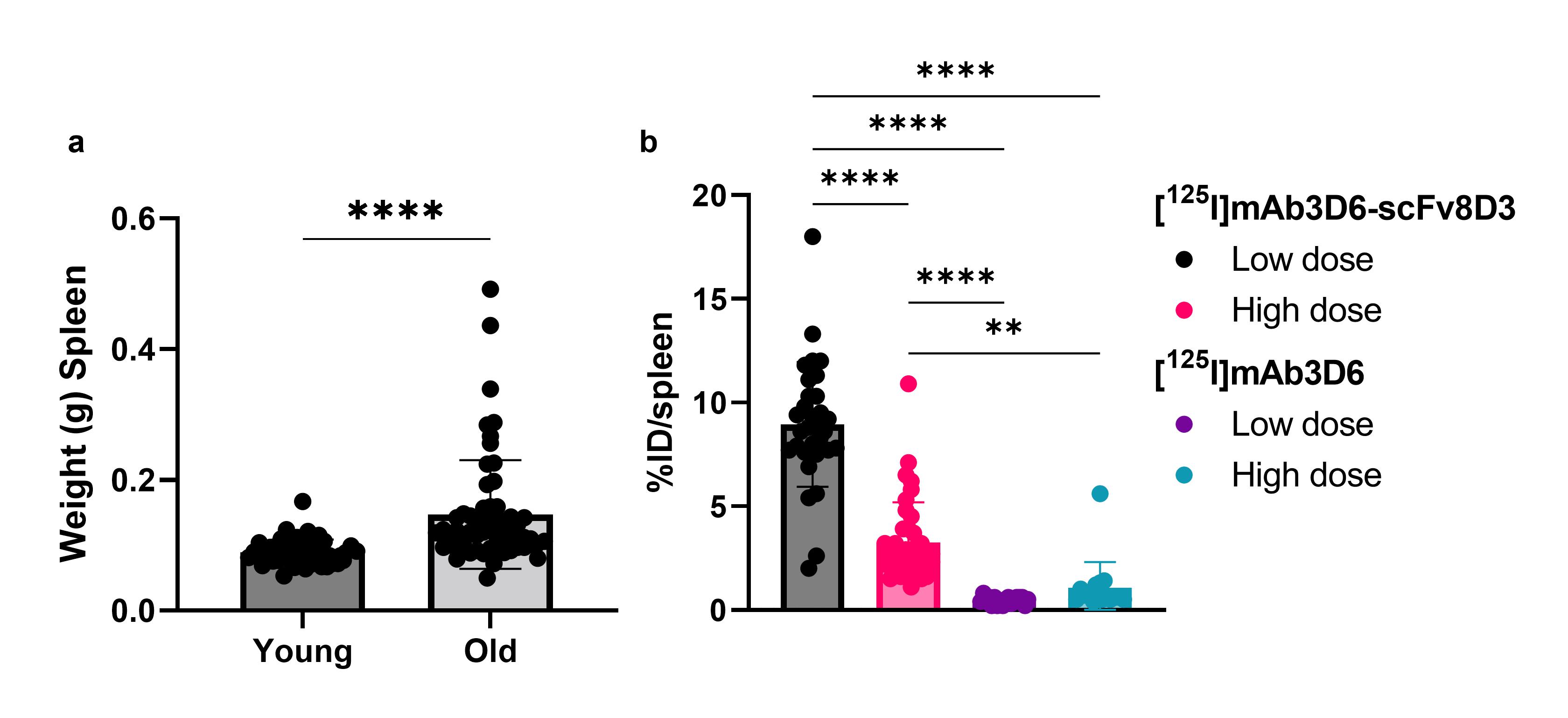


**Fig. S2 a** Weight of spleen in young and aged mice **b** %ID/spleen of bispecific or regular antibody at high and low doses, differences were analysed by one-way ANOVA with Bonferroni’s posthoc test.


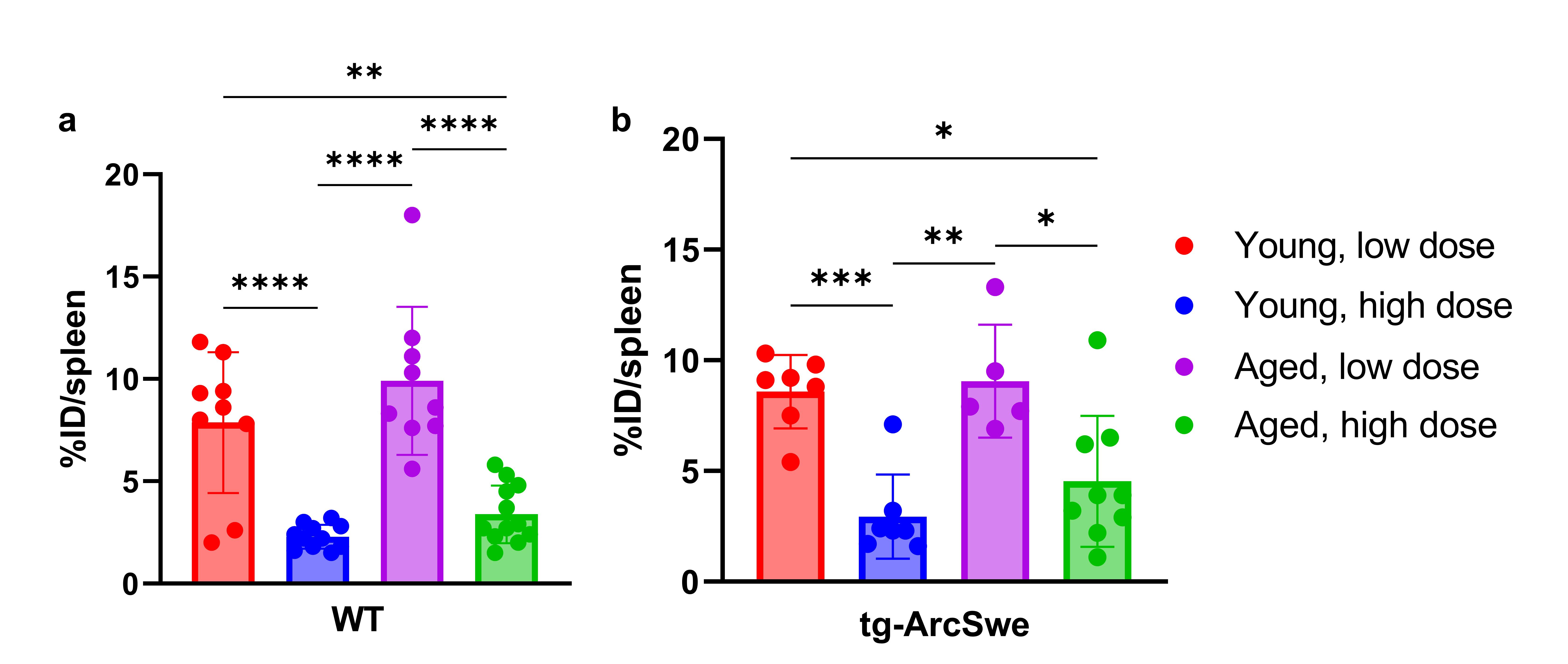


**Fig. S3 a** %ID/spleen of [^125^I]mAb3D6-scFv8D3 in young and aged, and low and high doses in **a** WT and **b** tg-ArcSwe mice, differences were analysed by one-way ANOVA with Bonferroni’s posthoc test.

**Brain**


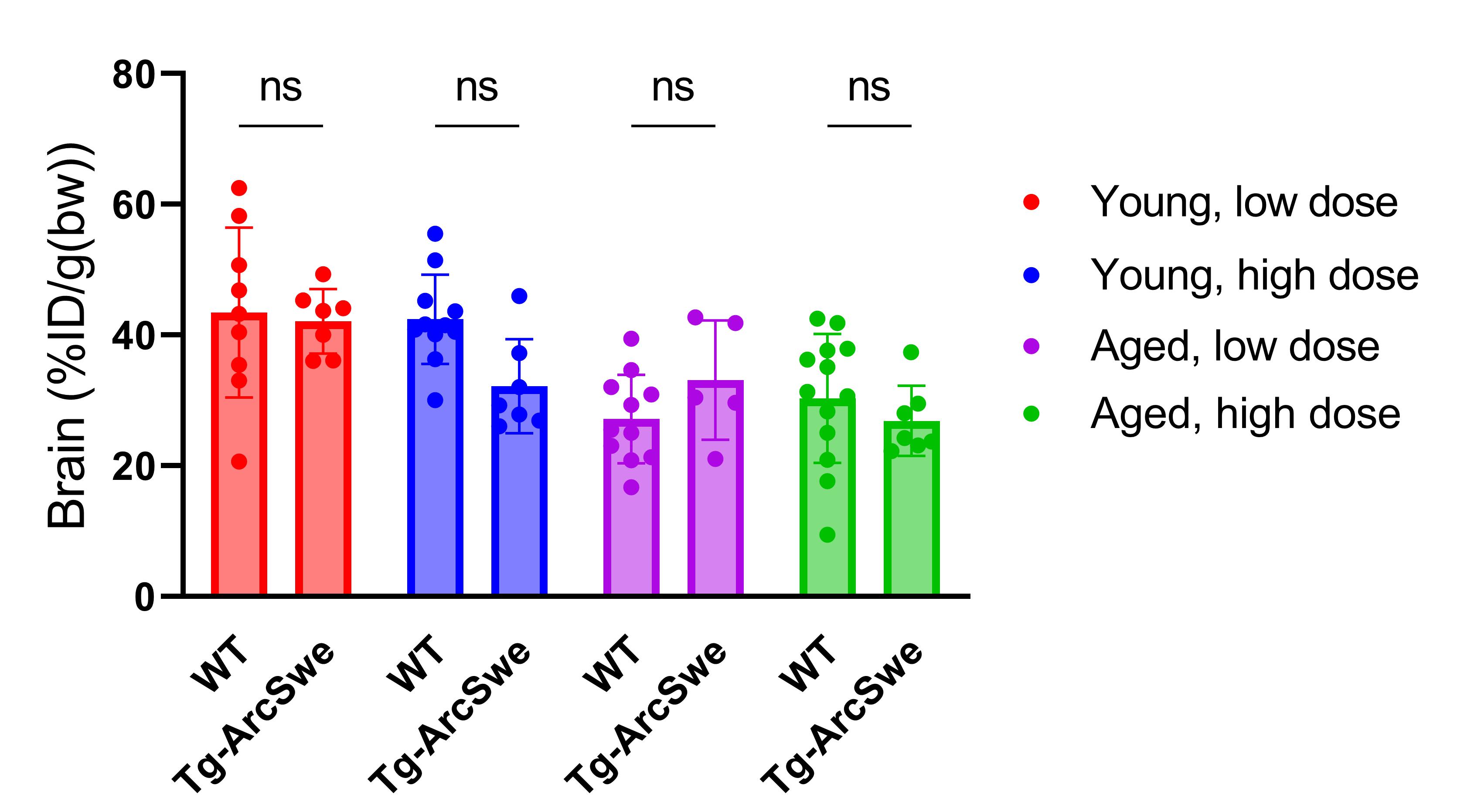


**Fig S4** %ID/g/bw brain of [^125^I]mAb3D6-scFv8D3. No difference between WT and tg-ArcSwe in brain uptake after 2 h. Data was analysed with pairwise two-way ANOVA with Bonferroni posthoc test.


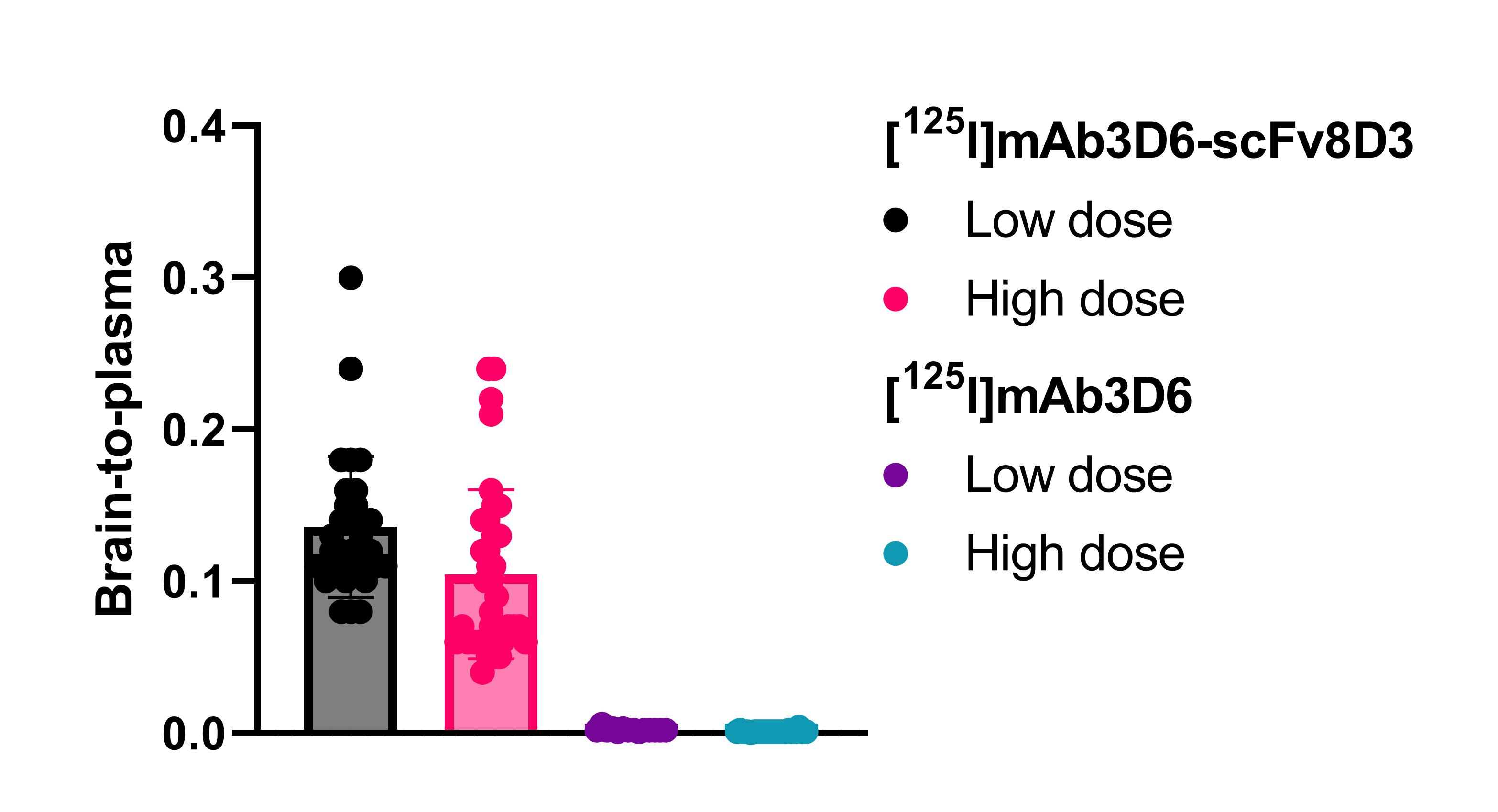


**Fig. S5** Brain-to-plasma ratios for [^125^I]mAb3D6-scFv8D3 or [^125^I]mAb3D6 -injected mice at low or high dosing.

**Capillary depletion**


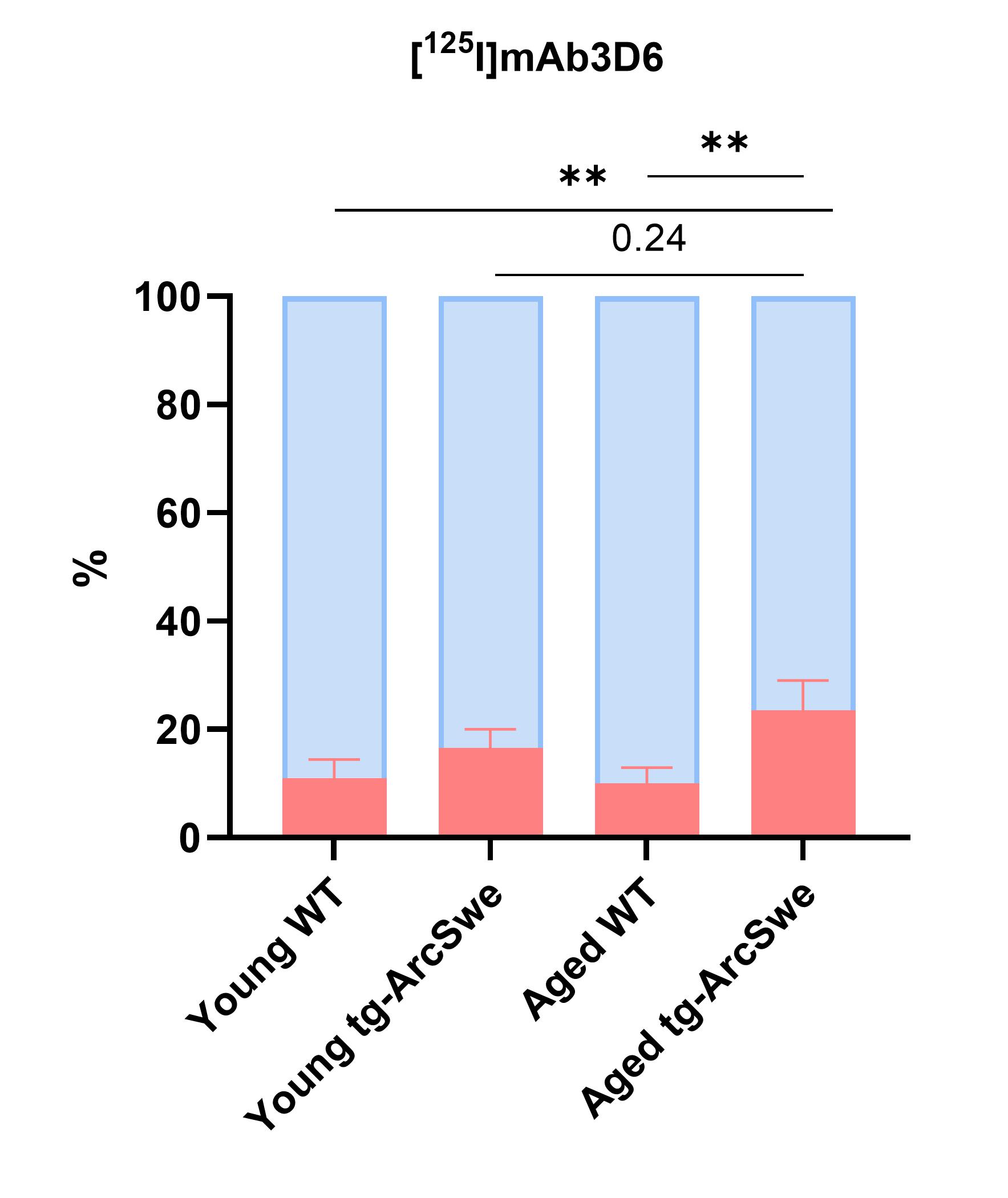


**Fig. S6** Capillary depletion for [^125^I]mAb3D6-administered mice at high dose, expressed as percentage in parenchymal (light blue) or capillary enriched (light red) fractions. Young WT n= 3, Young tg-ArcSwe n = 2, Aged WT n = 4, Aged tg-ArcSwe n= 4. Two-way ANOVA with Bonferroni correction for multiple comparisons.


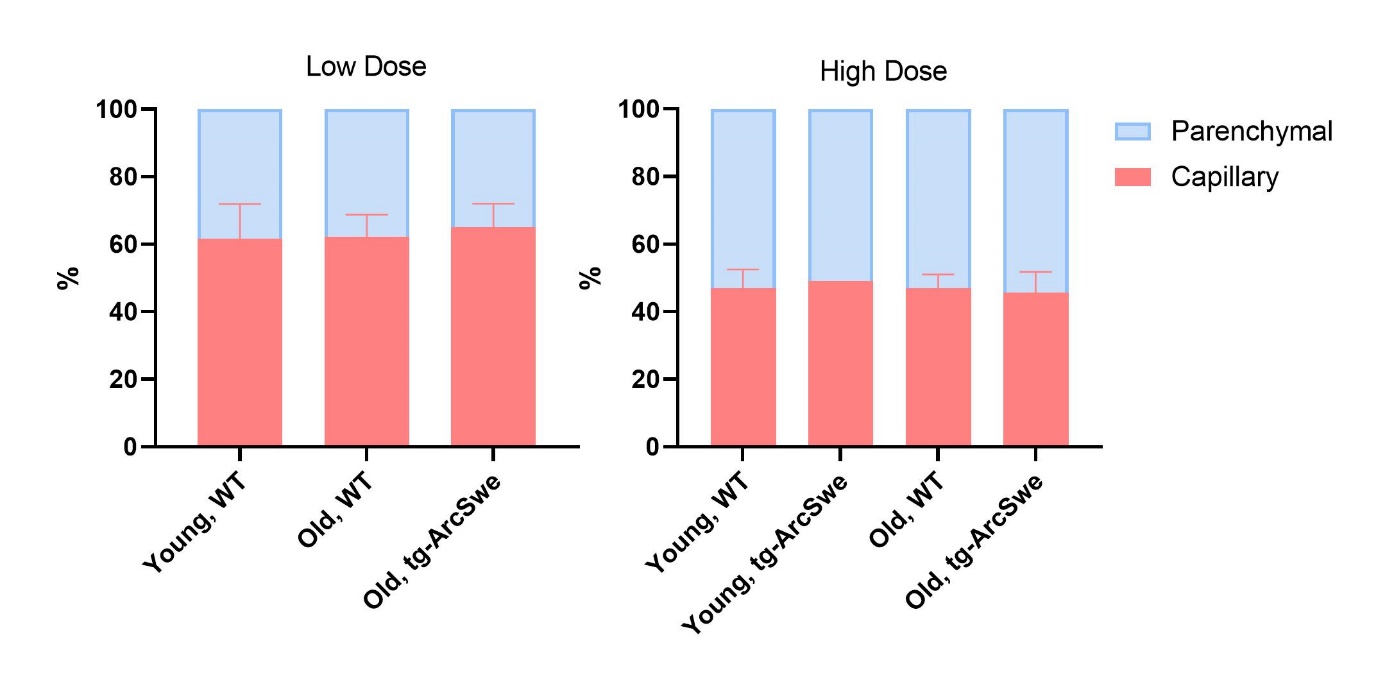
**Fig. S7 a** Capillary depletion for all low [^125^I]mAb3D6-scFv8D3-dosed mice (n= 3,4,7), and **b** all high dosed mice (n=3,3,3,1), expressed as percentage in parenchymal (light blue) or capillary enriched (light red) fractions.


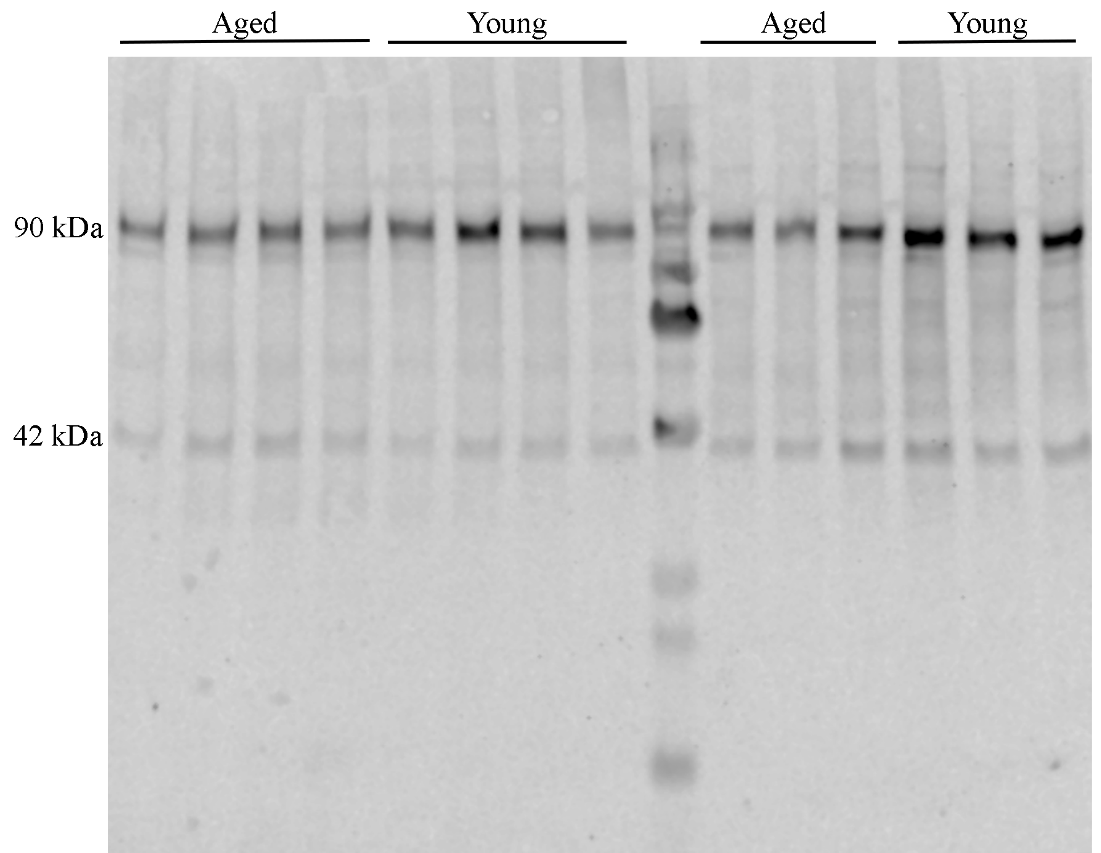

**Fig. S8** Full membrane of capillary enriched brain pellets detected with anti-TfR1 antibody for TfR1 (90kDa), and anti-β-actin, β-actin (42kDa) as loading control.
