# Supplementary figures and images for: Age, dose, and binding to TfR on blood cells influence brain delivery of a TfR-transported antibody
Source: Fluids Barriers CNS. 2023 May 11;20:34. doi: 10.1186/s12987-023-00435-2 (PMC10173660; doi:10.1186/s12987-023-00435-2)

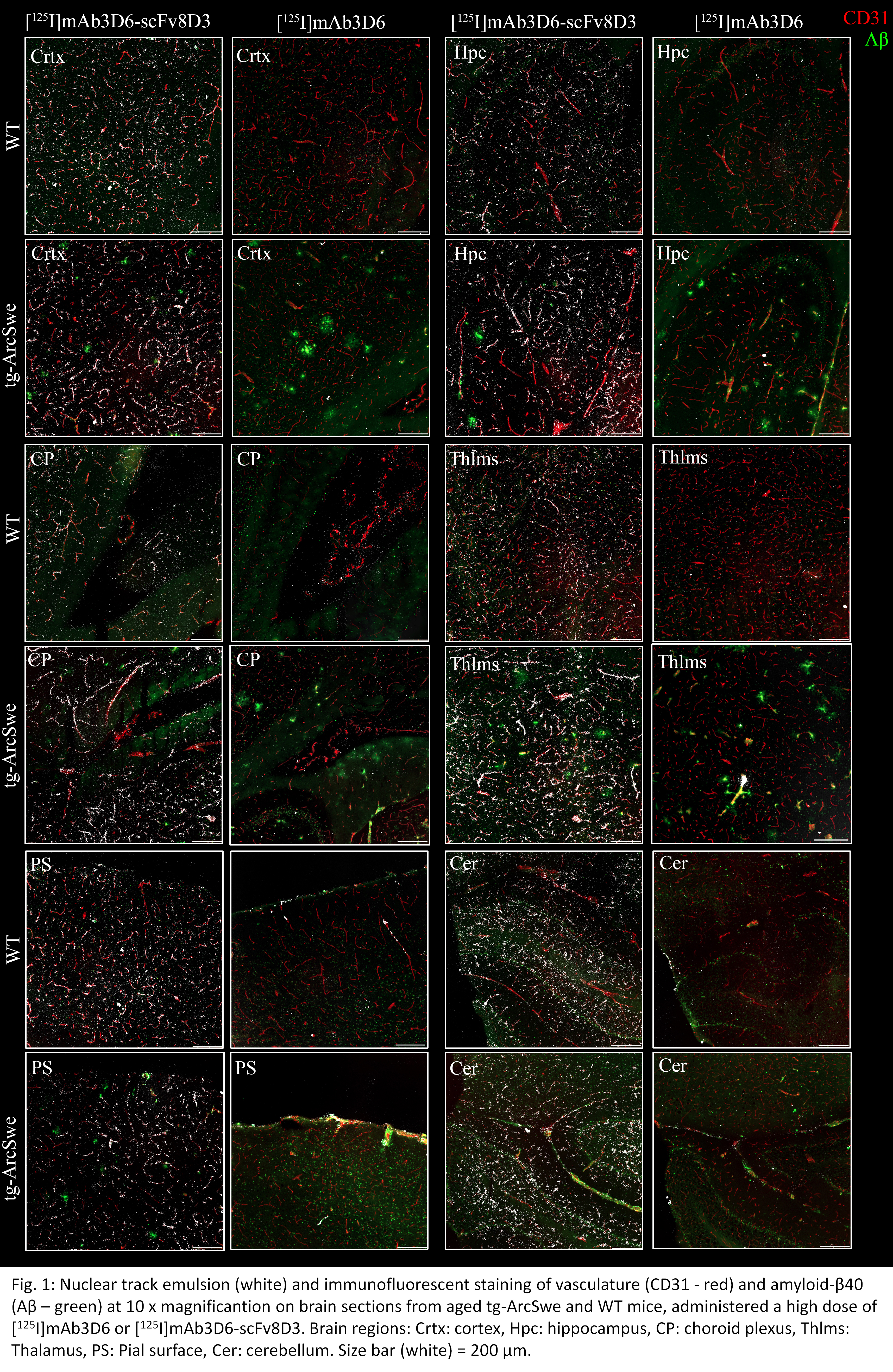

Supplement: Supplementary file 2 — Additional file 2: Figure 1. Nuclear track emulsion at different brain regions in [125I]mAb3D6-scFv8D3 or [125I]mAb3D6-injected mice. [file 12987_2023_435_MOESM2_ESM.jpg]
